# Universal activity-based labeling method for ammonia- and alkane-oxidizing bacteria

**DOI:** 10.1038/s41396-021-01144-0

**Published:** 2021-11-06

**Authors:** Dimitra Sakoula, Garrett J. Smith, Jeroen Frank, Rob J. Mesman, Linnea F. M. Kop, Pieter Blom, Mike S. M. Jetten, Maartje A. H. J. van Kessel, Sebastian Lücker

**Affiliations:** 1 Department of Microbiology, RIBES, Radboud University, Heyendaalseweg 135, 6525 AJ Nijmegen, the Netherlands; 2 Soehngen Institute of Anaerobic Microbiology, Radboud University, Heyendaalseweg 135, 6525 AJ Nijmegen, the Netherlands; 3 Present Address: Division of Microbial Ecology, Center for Microbiology and Environmental Systems Science, University of Vienna, Althanstraße 14, 1090 Vienna, Austria

**Keywords:** Environmental microbiology, Sequencing, Microbiology

## Abstract

The advance of metagenomics in combination with intricate cultivation approaches has facilitated the discovery of novel ammonia-, methane-, and other short-chain alkane-oxidizing microorganisms, indicating that our understanding of the microbial biodiversity within the biogeochemical nitrogen and carbon cycles still is incomplete. The in situ detection and phylogenetic identification of novel ammonia- and alkane-oxidizing bacteria remain challenging due to their naturally low abundances and difficulties in obtaining new isolates from complex samples. Here, we describe an activity-based protein profiling protocol allowing cultivation-independent unveiling of ammonia- and alkane-oxidizing bacteria. In this protocol, 1,7-octadiyne is used as a bifunctional enzyme probe that, in combination with a highly specific alkyne-azide cycloaddition reaction, enables the fluorescent or biotin labeling of cells harboring active ammonia and alkane monooxygenases. Biotinylation of these enzymes in combination with immunogold labeling revealed the subcellular localization of the tagged proteins, which corroborated expected enzyme targets in model strains. In addition, fluorescent labeling of cells harboring active ammonia or alkane monooxygenases provided a direct link of these functional lifestyles to phylogenetic identification when combined with fluorescence in situ hybridization. Furthermore, we show that this activity-based labeling protocol can be successfully coupled with fluorescence-activated cell sorting for the enrichment of nitrifiers and alkane-oxidizing bacteria from complex environmental samples, enabling the recovery of high-quality metagenome-assembled genomes. In conclusion, this study demonstrates a novel, functional tagging technique for the reliable detection, identification, and enrichment of ammonia- and alkane-oxidizing bacteria present in complex microbial communities.

## Introduction

Autotrophic ammonia- and methane-oxidizing bacteria (AOB and MOB, respectively) [1] are ubiquitous in the environment [2] and are of high biotechnological interest [3–9]. Besides their similar environmental distributions, they share many biochemical, morphological, and physiological characteristics [10]. More specifically, both microbial guilds can perform aerobic oxidation of ammonia and methane due to the substrate promiscuity of their key enzymes, but neither group exhibits growth on the alternative substrate [10–12]. Over the last two decades, metagenomic approaches resulted in the identification of novel groups of ammonia- [13–15] and methane-oxidizing microorganisms [2, 16–18], highlighting that our understanding of the microbial biodiversity within the nitrogen and carbon biogeochemical cycles can still be expanded [19]. However, even when detected in metagenomic datasets, linking a function to a specific microorganism remains challenging, as it often requires tedious and intricate cultivation techniques to isolate these slow-growing and fastidious microorganisms. Thus, there is an urgent need for robust cultivation-independent methods that provide reliable information regarding the identity and activity of the microorganisms present in complex microbial communities. To achieve this, various in vivo and in vitro activity-based protein profiling (ABPP) protocols have been developed to link detectable marker molecules to catalytically active enzymes with specific functions [20].

ABPP techniques employ bifunctional enzyme probes that feature (i) a reactive group, which covalently binds to the active site and thereby inhibits the enzyme, and (ii) an ethynyl or azide group that allows the attachment of a reporter group (e.g., fluorophores or biotin) to the enzyme via a Cu(I)-catalyzed alkyne-azide cycloaddition (CuAAC) reaction [21]. These protocols exhibit high specificity for the targeted enzymes as sufficient accessibility of the bifunctional enzyme probe to the active site and structural and/or chemical similarity with the substrate are necessary [20]. The subsequent use of rapid CuAAC reactions performed under mild, aqueous conditions guarantees minimal unspecific reactivity of the reporters and enables the use of low molecular weight bifunctional probes that can penetrate biological membranes [22–24]. Subsequently, depending on the reporter type, the reporter-conjugated enzyme can be subjected to numerous downstream applications such as fluorescent imaging [25–27], mass spectrometry-based proteomics [28, 29], and affinity purification of the labeled proteins [30]. Several microbial proteins have been studied using ABPP protocols so far. These studies provided significant insights into antibiotic resistance, enzymatic functions in pathogenic bacteria (e.g., serine proteases, kinases, ATPases, fatty acid synthases, glycoside hydrolases) and protein redox dynamics [20]. However, only a limited number of ABPP protocols targeting microbial proteins catalyzing key processes of biogeochemical cycles have been developed [14, 31–33].

Enzymes in the copper-containing membrane monooxygenase (CuMMO) family catalyze diverse reactions, including the oxidation of ammonia, methane, and simple short-chain hydrocarbons [34]. These CuMMO enzymes generally exhibit high genetic, structural, and catalytic similarities [35–37]. AOB and ammonia-oxidizing archaea (AOA) use ammonia monooxygenase (AMO) for the first step of nitrification, the oxidation of ammonia to hydroxylamine [38]. Similarly, the particulate methane monooxygenase (pMMO) present in most MOB oxidizes methane to methanol [39, 40]. Other hydrocarbon-oxidizing CuMMO (pHMO) enzymes have been reported to catalyze C_2_–C_4_ alkane oxidation in some, taxonomically diverse bacteria [34, 41, 42]. Moreover, some MOB also contain a cytoplasmic diiron-containing soluble methane monooxygenase (sMMO), which is expressed mainly under copper-limited conditions [43, 44] and can even constitute the sole methane monooxygenase in some MOB [45]. Although the substrate for both methane monooxygenases is identical, the sMMO shares little structural homology with pMMO or other CuMMO enzymes but rather exhibits a high degree of similarity to known soluble diiron alkane and other hydrocarbon monooxygenases (sHMO) [37, 46].

The recent discovery of the novel comammox (complete ammonia oxidation) *Nitrospira*, featuring a distinct AMO enzyme previously misclassified as an “unusual” pMMO [13, 14], exemplified our still incomplete understanding of the microbial diversity catalyzing ammonia, methane, and simple alkane oxidation. To date, many insights into the distribution, abundance, and activity of ammonia-oxidizing prokaryotes and MOB in natural environments have been obtained by employing reversible [47–50] and irreversible inhibitors [31, 51–53]. Many terminal *n-*alkynes can be catalytically activated by CuMMO enzymes producing reactive intermediates that bind covalently to the enzyme and act as mechanism-based irreversible inactivators [46, 52, 54–56]. Similarly, alkynes have been established as mechanism-based inactivators of sMMO and other sHMO [46, 56]. The alkyne 1,7-octadiyne (1,7OD) was recently characterized as a mechanism-based inactivator of the AMO enzyme in *Nitrosomonas europaea* and was successfully used in in vitro ABPP protocols [31].

In this study, we describe the use of 1,7OD in an activity-based labeling protocol for the in situ biotin or fluorescent labeling of bacterial ammonia, methane, and alkane monooxygenases. In combination with transmission electron microscopy, the biotin tagging of AMO and pMMO permitted the study of their intracellular distribution. Fluorescent labeling of diverse CuMMO and sHMO enzymes allowed the function-based detection of catalytically active ammonia-, methane-, and alkane-oxidizing microorganisms by fluorescence microscopy, which can be combined with fluorescence in situ hybridization [57] for phylogenetic identification. Moreover, this method can efficiently be used for fluorescence-activated cell sorting (FACS), allowing the enrichment of ammonia-, methane-, and alkane-oxidizing bacteria from complex environmental samples. Together with downstream metagenomics, this facilitates the targeted retrieval of high-quality metagenome-assembled genomes (MAGs) of functionally constrained subpopulations.

## Materials and methods

### Cultivation

A pure culture of the canonical AOB *N. europaea* (DSM 28437) was obtained from the Leibniz Institute DSMZ-German Collection of Microorganisms and Cell Cultures (Braunschweig, Germany). *N. europaea* was grown in batch in mineral salts (MS) medium amended with 10 mM NH_4_Cl [58]. A culture of the AOB *Nitrosospira multiformis* was maintained in MS medium supplemented with 1 mM ammonium as described by Norton et al. [59]. The comammox strain *Nitrospira inopinata* was grown as described elsewhere with 1 mM NH_4_Cl [60], and the canonical nitrite-oxidizing bacterium *Nitrospira moscoviensis* M-1 in MS medium according to Mundiger et al. [61]. Cultures of the MOB *Methylotetracoccus oryzae* (nitrogen-fixing, Type Ib methanotroph), *Methylosinus sporium* M29 (Type II methanotroph), *Methylocella tundrae* (Type II methanotroph containing sMMO only), and *Methylacidiphilum fumariolicum* (Type III methanotroph) were maintained in batch in nitrate mineral salts (NMS) medium as previously described [17, 62–64]. The propane-oxidizing pHMO-containing *Rhodococcus* sp. strain ZPP was grown in NMS medium [41] and the butane-degrading sHMO-containing *Thauera butanivorans* strain Bu-B1211 (DSM 2080) [65] in Brunner mineral medium (DSMZ medium 457). All alkane-oxidizing cultures were inoculated into 20 mL medium in 120 mL serum bottles and the headspace was amended with 1% (v/v) of methane, propane, or butane, respectively, which were replenished when consumed. A pure culture of the diazotrophic *Kyrpidia spormannii* FAVT5 was cultivated according to Hogendoorn et al. [66]. *Escherichia coli* was grown in standard Luria–Bertani broth. An enrichment culture containing the comammox species *Ca*. Nitrospira nitrosa and *Ca*. Nitrospira nitrificans was maintained in a sequencing batch bioreactor as reported previously [14]. An enrichment culture of the novel comammox species *Ca*. Nitrospira kreftii was maintained in a membrane chemostat as described elsewhere [67]. A nitrifying enrichment culture containing canonical AOB, canonical and comammox *Nitrospira* was maintained in a continuous membrane bioreactor fed with MS medium (200 mg/L MgSO_4_⋅7H_2_O, 30 mg/L CaCl_2_⋅2H_2_O, 58 mg/L NaCl) supplemented with 0.5 mL/L of a trace element stock solution composed of NTA (15 g/L), ZnSO_4_⋅7H_2_O (0.43 g/L), CoCl_2_⋅6H_2_O (0.24 g/L), MnCl_2_⋅4H_2_O (0.99 g/L), CuSO_4_⋅5H_2_O (0.25 g/L), Na_2_MoO_4_⋅2H_2_O (0.22 g/L), NiCl_2_⋅6H_2_O (0.19 g/L), NaSeO_4_·10H_2_O (0.021 g/L), H_3_BO_4_ (0.014 g/L), CeCl·6H_2_O (0.24 g/L), and 0.5 mL/L of an iron stock solution composed of NTA (10 g/L) and FeSO_4_ (5 g/L). The pH was automatically maintained at 8.0 by dosing 1 M KHCO_3_. The culture was grown under substrate-limited conditions with <100 μΜ ammonium, <20 μΜ nitrite, and 250–500 μΜ nitrate supplied to the system. Activated sludge samples were obtained from the aeration tank of a municipal wastewater treatment plant (WWTP) in Groesbeek, The Netherlands (51°45'34.3“N 5°57'15.9“E).

Unless stated otherwise, biomass from the different pure cultures, bioreactors, and the WWTP sample was harvested by gentle centrifugation (600 × *g* for 10 min), washed twice, and subsequently resuspended in MS medium to a final density corresponding to approximately 100 μg protein/mL.

### Inactivation of comammox *Nitrospira* by 1,7OD

To test for the irreversible inactivation of comammox *Nitrospira* by 1,7OD, biomass from the *Ca*. N. kreftii enrichment culture [67] was harvested and resuspended in 50 mL MS medium without nitrogen containing 100 μΜ 1,7OD (99% purity, Merck KGaA, Darmstadt, Germany) from a stock solution (75.4 mM) in dimethyl sulfoxide (DMSO). Suspensions were incubated for 10 min in the dark in a shaking incubator (150 rpm, 24 °C). Subsequently, incubations were supplemented with NH_4_Cl to a final concentration of 350 μM and were incubated for 72 h. Control incubations receiving only DMSO were performed in parallel as a negative control. As a positive control for the inhibition of the ammonia oxidation activity, cells were incubated in the presence of 100 μM allylthiourea (ATU; ≥98% purity, Merck KGaA, Darmstadt, Germany), which has been shown to inhibit comammox *Nitrospira* previously [14]. Finally, the specificity of 1,7OD as inhibitor of the ammonia-oxidizing activity was verified by incubating comammox *Nitrospira* cells in the presence of 100 μM 1,7OD and 20 μM NaNO_2_. All incubations were performed in three biological replicates. During incubation, samples for the determination of ammonium, nitrite, and nitrate concentrations were taken every 2 h for the first 8 h of incubation, as well as after 24, 48, and 72 h of incubation.

### Analytical methods

Ammonium concentrations were measured fluorometrically using a modified orthophatal-dialdehyde assay [68]. Nitrite and nitrate concentrations were determined colorimetrically with the Griess reaction [69] as described elsewhere [70]. For the determination of total protein content, cells were lysed using the Bacterial Protein Extraction Reagent (Thermo Fisher Scientific, Waltham, MA, USA) according to the manufacturer’s instructions in combination with mild sonication (1 min, 20 Hz). Post lysis, protein concentrations were measured using the Pierce bicinchoninic acid protein assay kit (Thermo Fisher Scientific, Waltham, MA, USA) according to the “Enhanced Test-tube” protocol. All fluoro- and colorimetric measurements were performed using a Tecan Spark M10 plate reader (Tecan Trading AG, Männedorf, Switzerland). Methane, propane, and butane concentrations were measured using gas chromatography (HP 5890a, equipped with a flame ionization detector and a Porapak Q column at 80 °C).

### In situ activity-based fluorescent labeling of ammonia and alkane monooxygenase-containing cells

Active biomass of the different pure and enrichment cultures, as well as the WWTP sample (see above) was harvested as described above, resuspended in 50 mL MS medium and incubated with 100 μM 1,7OD for 30 m (150 rpm) in the dark. Subsequently, cells were pelleted by gentle centrifugation (600 × *g* for 10 min), washed twice in sterile PBS, pH 7.5 and fixed using a 50% (v/v) ethanol/PBS solution for 10 min at room temperature (RT). Fixed biomass was washed once with PBS and subjected to the CuAAC reaction, which was performed in plastic microcentrifuge tubes (1.5 mL) in a final volume of 250 μL. Biomass was resuspended in 221 μL sterile PBS, mixed with 12.5 μL of a 100 mM freshly prepared sodium ascorbate solution (≥99% purity, Merck KGaA, Darmstadt, Germany) and 12.5 μL of a 100 mM freshly prepared aminoguanidine hydrochloride solution (≥98% purity, Merck KGaA). A dye mixture containing 1.25 μL of a 20 mM CuSO_4_ solution (99.99% purity, Merck KGaA), 1.25 μL of a 100 mM Tris (3-hydroxypropyltriazolylmethyl)amine solution (95% purity, Merck KGaA), and 0.3 μL of 5 mM Azide-Fluor 488 in DMSO (≥90% purity, Merck KGaA) was incubated in the dark for 3 min. Subsequently, the dye mixture (2.8 μL) was added to the CuAAC reaction tubes. The tubes were gently mixed and incubated for 60 min (RT, in the dark). CuAAC reactions were terminated by harvesting the cells by gentle centrifugation (600 × *g* for 10 min). Cell pellets were washed three times with sterile PBS to remove unbound fluorophores and either used directly for downstream analyses, or resuspended in a 50% (v/v) ethanol/PBS solution and stored at −20 °C.

### Fluorescence in situ hybridization and microscopy

Activity-based labeled biomass was hybridized with fluorescently tagged oligonucleotides as described by Daims et al. [71]. Probes used in this study (Supplementary Table S1) were 5’ or 5’ and 3’-labeled with the dyes Cy3 or Cy5 [72]. After hybridization and washing, slides were dried and embedded in DAPI-containing Vectashield antifading mounting medium (#H-1200, Vector Laboratories Inc., Burlingame, CA). Probe and activity-conferred fluorescence was recorded using a Leica TCS Sp8x confocal laser microscope (CLSM; Leica Microsystems B.V., Amsterdam, the Netherlands) equipped with a 405 nm UV diode and a pulsed white light laser. Images were recorded using 63× or 100× oil immersion objectives at a resolution of 1024 × 1024 pixels and 8-bit depth.

The spatial distribution of the activity-based fluorescent signal within bacterial cells was investigated using the HyVolution deconvolution module of the Huygens Essential Suite (Scientific Volume Imaging B.V, Hilversum, The Netherlands). *N. europaea*, *N. inopinata*, *M. oryzae*, and *M. tundarae* were used as representatives of canonical AOB, comammox bacteria, and pMMO and sMMO-only containing MOB, respectively. Images were acquired using a Leica Sp8x CLSM with a 100× oil immersion objective, a 0.5 AU pinhole size at a resolution of 1024 × 1024 pixels, and were deconvolved using the resolution-optimized algorithm of the HyVolution module.

### Immunogold localization of the AMO and pMMO enzymes and quantification

Active cells of *N. europaea* and *M. oryzae* were inactivated using 1,7OD as described above, harvested, fixed for 30 min at RT (2% PFA, 0.5% GA in 0.1 M phosphate buffer pH 7) and processed for Tokuyasu sectioning [73, 74]. Cells incubated in the presence of DMSO without 1,7OD were used as a negative control. The blocks were trimmed to provide a suitable block face. Subsequently, 65-nm sections were cut on a 35° diamond knife (Diatome, Biel/Bienne, Switzerland) at −100 °C in a cryo-ultramicrotome (UC7/FC7 Leica Microsystems, Vienna, Austria), picked up using a mixture of 1% methylcellulose in 1.2 M sucrose [75] and transferred to 100#H copper grids (Agar Scientific LTD., Essex, UK).

In order to successfully localize the AMO/pMMO enzymes in the bacterial cells, the thawed cryo-sections were subjected to a CuAAC reaction (as described above) using 5 mM biotin-azide in DMSO (>95% purity, Jena Bioscience GmbH, Jena, Germany) instead of Azide-Fluor 488. Subsequently, immunogold localization of the biotinylated enzymes was performed according to Slot and Geuze (2007) using a rabbit anti-biotin antibody (ab53494, Abcam plc., Cambridge, UK, diluted 1:600 in 1% BSA-C) and 10 nm protein A gold (CMC-UMC Utrecht, the Netherlands). Sections were embedded in methylcellulose containing 0.2% uranyl-acetate before imaging using a JEOL JEM-1400 flash transmission electron microscope, operating at 120 KV.

The localization of the immunogold labels was counted in 9–12 cells for each sample and was classified in one out of four classes: (i) resin, (ii) membrane-associated, (iii) cytoplasmic, and (iv) unclear. To this end, circles with a diameter of 15 nm were drawn around the center of each gold particle. The gold particles themselves had a given diameter of 10 nm and the antibody-protein A complex accounted for another 10 nm; thus, the epitopes the antibodies were bound to were expected to lie somewhere within these 15 nm circles. In case gold particles were located in the cytoplasm but with a membrane present within the circle, the label localization was classified as unclear.

The location of the epitopes that classified as “membrane-associated” was investigated in greater detail. More specifically, labels on the periplasmic side of the intracytoplasmic membrane (ICM) compartments were classified as “periplasmic” while labels present on the cytoplasmic side were classified as “cytoplasmic”. Labels localized on transversely sectioned membrane stacks or exactly on top of membranes were classified as unclear.

### Effect of growth stage on activity-based fluorescent labeling efficiency

Cells of *N. europaea* and *N. multiformis* were grown as described above in buffered medium (4.8 g/L HEPES) at pH 8.0 and 7.8, respectively. Control incubations with *N. europaea* and *N. multiformis* cells incubated in medium without ammonium as well as abiotic controls were performed in parallel. All incubations were performed in two biological replicates. Samples (10 mL) were taken regularly to monitor protein content, ammonium concentration, and pH. Additional samples (50 mL) were subjected to the activity-based labeling protocol as described above. For the quantification of the relative biovolume fractions that depicted AMO labeling, as well as of signal intensity, the activity-based labeling protocol was combined with FISH using an equimolar mixture of the probes Nso190, Nso1225 and NEU653 (Cy3) and EUB338mix (Cy5) (Supplementary Table S1). Subsequently, 15 image-pairs were recorded per sample at random fields of view using a Leica TCS Sp8x CLSM with the same laser intensity, gain (%) and pinhole settings. The images were imported into the image analysis software daime [76] and evaluated as described elsewhere [77].

### Targeted cell sorting of ammonia and alkane monooxygenase-containing bacterial cells

Biomass from the nitrifying enrichment culture and activated sludge from a municipal WWTP were subjected to the activity-based fluorescent labeling protocol as described above. For both biomasses, three types of control incubations were performed in parallel. These contained (i) incubation with 1,7OD, but no addition of the fluorescent azide-labeled dye in the CuAAC reaction, (ii) DMSO without 1,7OD during the initial incubation, with complete subsequent CuAAC reaction, and (iii) DMSO without 1,7OD during the initial incubation and no addition of the fluorescent azide-labeled dye in the CuAAC reaction. After labeling, large biomass particles and flocs were partially dispersed by mild sonification (30 s at 20 Hz) and either stored at –80 °C in glycerol-TE (GlyTE) buffer (10 mM Tris-HCl, 1 mM EDTA pH 8.0, 5% v/v glycerol), or processed directly. If frozen, samples were thawed on ice and subsequently subjected to FACS (BD FACSMelody, BD Biosciences, NJ, USA). Based on the initial cell density determined by FACS, samples were diluted up to 1:4 with sterile PBS to ensure a final event rate of <10,000 events/s. Control incubations, particularly the treatment with dye but without 1,7OD, were used to determine a gating strategy for removal of artifacts to exclude populations with off-target labeling or passive dye accumulation. Subsequently, DNA was extracted from the bulk-sorted cell clusters, the unsorted control incubations and the initial biomass, followed by metagenomic sequencing.

In addition, we tested for potential biases on the community composition caused by physical and chemical treatment steps that are part of the activity-based protocol. These included potential effects of washing steps, biomass fixation, sonication, and storing conditions. For this purpose, biomass from the nitrifying enrichment culture was subjected to different combinations of physical and chemical treatments occurring during the activity-based staining protocol (Supplementary Table S2), followed by DNA isolation and metagenomic sequencing (see below).

### DNA extraction and metagenomic sequencing

Total DNA from all samples was extracted using the DNeasy Blood & Tissue Kit (Qiagen Ltd., West Sussex, UK) according to the manufacturer’s instructions. Genomic sequencing libraries were prepared using the Nextera XT Kit (Illumina, San Diego, CA, USA) following the manufacturer’s recommendations using a total of 1 μg of input DNA, normalized to a concentration of 0.2 ng/µL. The libraries were sequenced on a MiSeq (Illumina, San Diego, CA, USA) with MiSeq Reagent Kit v.3 (2 × 300 bp, Illumina) according to the manufacturer’s instructions.

### Metagenome assembly and binning

Quality-trimming, adapter removal, and contaminant filtering of paired-end sequencing reads for all datasets were performed using BBDuk (BBTools version 37.76; with settings ktrim = r, k = 23, mink = 11, hdist = 1, qtrim = rl, trimq = 17, maq = 20, maxns = 0, minlen = 150) [78]. Processed reads from all libraries from the same biological sample were de novo co-assembled using metaSPAdes v3.11.1 [79] with default settings, which iteratively assembled the metagenome using *k*-mer sizes of 21, 33, 55, 77, 99, and 127 bp. After assembly, reads were mapped back to the metagenomic contigs for each sample separately using Burrows–Wheeler Aligner (version 0.7.17) [80], employing the “mem” algorithm. The mapping files were processed and converted using SAMtools version 1.9 [81]. Metagenomic binning was performed including all contigs ≥1500 bp, using differential coverage, sequence composition, and linkage information. To optimize the binning results, five different binning algorithms were used: BinSanity v0.2.6.1 [82], COCACOLA [83], CONCOCT [84], MaxBin 2.0 v2.2.4 [85], and MetaBAT 2 v2.12.1 [86]. The five bin sets were supplied to DAS Tool v1.0 [87] for consensus binning. Bin quality and completeness were assessed based on single-copy marker gene count by CheckM v1.0.7 [88]. Phylogenetic assignment of the final bins was predicted using the classification workflow of GTDB-Tk v0.2.1 [89].

Besides the assembly and automated binning method described above, the sequencing data retrieved from the FACS-sorted activated sludge sample were assembled separately, with subsequent binning of all contigs ≥1500 bp and manual refinement using the anvi’o metagenomics workflow [90]. Here, contigs not clustering with the rest of the bin, based on sequence composition or coverage, were inspected individually in the assembly graph using Bandage v0.8.1 [91]. In addition, genes present on these contigs were compared against the NCBI non-redundant nucleotide database using BLASTx [92]. Contigs without connections in the assembly graph and containing genes not matching the taxonomic affiliation of the bin were removed from the respective MAG. DRAM was used to annotate these MAGs and assess their quality to conform to standards [93]. MAGs were clustered with the automated bins using dRep (v2.0.0.) with a 99% identity cutoff [94].

### Marker gene recovery and read recruitment

Genes on assembled contigs ≥1500 bp were identified by Prodigal [95]. The translated amino acid sequences were mined using hidden Markov models (HMM) available from the Pfam database to extract the assembled genes for AMO subunit A (*amoA*, PF02461) and RNA polymerase beta subunit (*rpoB*, PF04563) [96, 97]. Genes with a sequence score less than 20 or an *e* value greater than 1e–5 were inspected manually to remove false-positives. Amino acid sequences of identified CuMMO genes were aligned with the full-length representatives used to build the HMM using MAFFT (v7.397) [98]. The alignment was manually modified to remove partial sequences, including some references, and gapped regions at both ends were trimmed to the first position at which all references contained a residue, resulting in 113 sequences (98 references) and 227 total alignment positions. Phylogenetic reconstruction of was performed using RAxML (v8.2.10) [99] with rapid bootstrapping (100 bootstraps) and determining the best-scoring maximum-likelihood tree using the Gamma matrix and GTR substitution model. For both marker gene sets, corresponding nucleotide sequences >50% of the gene length were used to quantify read recruitment using BBMap (BBTools version 37.76 idfilter = 0.99, pairedonly = t, ambiguous = random) [78]. Read recruitment per gene and sample was normalized by gene length and sequencing depth as paired reads per kilobase gene length per million reads mapped to the full assembly. R was used to normalize read abundances, calculate correlations, and for visualization [100, 101].

## Results

### Inhibition of ammonia oxidation in comammox by 1,7OD

The ability of 1,7OD to act as an irreversible and mechanism-based inactivator of the AMO enzyme in the canonical AOB *N. europaea* was previously demonstrated [31]. Similarly, diverse CuMMOs and sHMOs including methane monooxygenases were shown to be partially inhibited by alkynes [46, 56, 102]. However, comammox *Nitrospira* harbor a phylogenetically distinct AMO enzyme [13, 14, 36]. Thus, to test the efficacy of inactivation of the comammox AMO by 1,7OD, inhibition of ammonia oxidation activity was determined in the *Ca*. N. kreftii enrichment culture, which contained comammox *Nitrospira* as the sole ammonia-oxidizing microorganism [67].

**Fig. 1:**
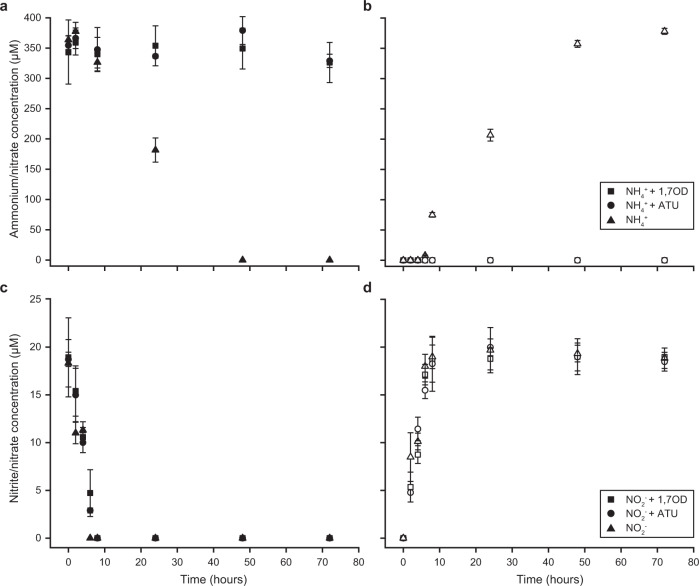
Inhibition of ammonia oxidation activity of the comammox organism *Ca*. N. kreftii by 1,7OD. **a** Ammonium consumption and **b** nitrate production in the presence of 350 μM NH_4_^+^ with and without the addition of 100 μΜ 1,7OD or ATU. **c** Nitrite consumption and **d** nitrate production in the presence of 20 μM NO_2_^-^ with and without the addition of 100 μΜ 1,7OD or ATU. Concentrations of the substrates ammonium (**a**) and nitrite (**c**) are indicated by solid, produced nitrate (**b**, **d**) by open symbols. Data points represent the mean of technical triplicates, error bars the standard deviations between three biological replicates.

In the absence of 1,7OD, the culture oxidized 350 μM ammonium in 48 h to nitrate without intermediate nitrite accumulation. In contrast, addition of 100 μΜ 1,7OD resulted in a rapid and complete inhibition of the ammonia-oxidizing activity, which was comparable to the inhibitory effect of 100 μM ATU (Fig. 1a, b). No influence of 1,7OD or ATU on the nitrite-oxidizing activity of the culture was detected (Fig. 1c, d).

### In situ fluorescent labeling of ammonia and alkane monooxygenases

**Fig. 2:**
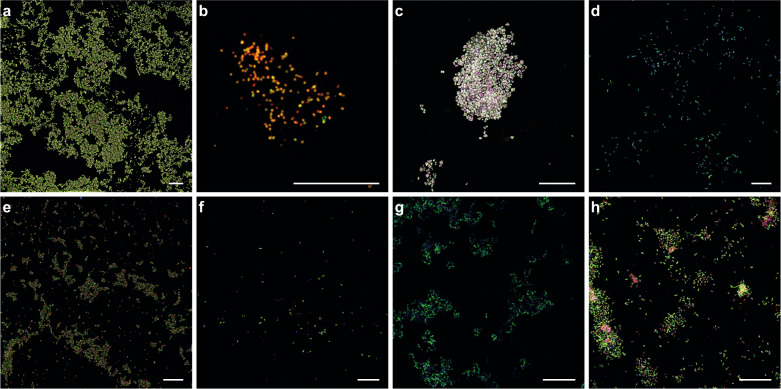
Activity-based fluorescent labeling of catalytically active ammonia-, methane-, and other alkane-oxidizing microorganisms. Fluorescent micrographs of 1,7OD pre-incubated cells of **a** *Nitrosomonas europaea*, **b** *Nitrospira inopinata*, **c** *Methylotetracoccus oryzae*, **d** *Methylosinus sporium* M29, **e** *Methylacidiphilum fumariolicum* SolV, **f** *Methylocella tundrae*, **g** *Rhodococcus* sp. strain ZPP, and **h** *Thauera butanivorans*. Cells were stained with the activity-based labeling protocol (shown in green) and FISH probes specific for **a** AOB (Nso190, Neu653, Nso1225; red), **b** *Nitrospira* (Ntspa662, Ntspa712; red), **c, f** Gammaproteobacteria (Gam42a; red), **d** Alphaproteobacteria (Alf0001b, Alf0968; red), **e** Verrucomicrobia (EUB338III; red), **h** Betaproteobacteria (Bet42a; red), and **a**–**f**, **h**) all bacteria (EUB338mix; blue). Cells in **g** were counterstained with DAPI (blue). Scale bars correspond to 20 µm.

We adapted the activity-based AMO labeling protocol developed by Benett et al. [31] to allow the in situ fluorescent labeling of ammonia-, methane-, and other alkane-oxidizing bacteria. Biomass of different CuMMO or sHMO-containing microorganisms was incubated with 100 μM 1,7OD, which leads to the formation of stable 1,7OD-enzyme complexes within the bacterial cells [31, 52]. After EtOH fixation, the inactivated biomass was subjected to a highly specific CuAAC reaction to enable the covalent coupling of the alkyne-labeled enzymes to an azide-labeled Fluor 488 dye, which resulted in the fluorescent labeling of AMO, pMMO, sMMO, pHMO, and sHMO-containing microorganisms (Fig. 2 and Supplementary Figs. S1 and S2).

The efficiency and specificity of the activity-based labeling was verified using pure cultures of the following nitrifying microorganisms: (i) *N. europaea* and (ii) *N. multiformis*, two well-studied canonical AOB; (iii) *N. inopinata*, the only comammox bacterium available as pure culture [60]; (iv) *N. moscoviensis*, a canonical nitrite-oxidizing bacterium; and (v) *Nitrosocosmicus franklandus*, an ammonia-oxidizing archaeon. Although in most cultures not all cells appeared to be stained, efficient staining of the *N. europaea*, *N. multiformis*, and *N. inopinata* cells was observed when 1,7OD-treated cells were subjected to the CuAAC reaction (Fig. 2a, b and Supplementary Fig. S2c). In contrast, no fluorescent signal was obtained when the cells were not treated with 1,7OD (Supplementary Fig. S1a, b), in canonical nitrite-oxidizing *N. moscoviensis* (Supplementary Fig. S1j), or in heterotrophic *E. coli* cells (Supplementary Fig. S1k). However, *N. franklandus* cells also did not show any fluorescent labeling (Supplementary Fig. S1i), indicating that the activity-based protocol was not able to label the archaeal AMO enzyme.

It has been reported that pMMOs and AMOs exhibit similar inhibition patterns [35] and that both particulate and soluble (p/s)MMO and p/sHMO enzymes can be inactivated by acetylene as well as other alkyne compounds [46, 52, 56, 103]. However, the effect of 1,7OD on methane- and other alkane-oxidizing enzyme activities has not been studied yet. To test whether the activity-based labeling protocol with 1,7OD could also label p/sMMOs and p/sHMOs, the following pure cultures were subjected to our labeling protocol: (i) *M. oryzae*, a type Ib methanotrophic gammaproteobacterium [64] and (ii) *M. sporium*, a type II methanotrophic alphaproteobacterium [62], both encoding pMMO; (iii) *M. fumariolicum*, an acidophilic methane oxidizer of the phylum *Verrucomicrobia* with multiple pMMO copies [17]; (iv) *M. tundrae*, an alphaproteobacterial methanotroph encoding only sMMO [63]; (v) *Rhodococcus* sp. strain ZPP, an ethane- and propane-oxidizing member of the *Actinobacteria* containing both pHMO and sHMO enzymes, which however was reported to use the pHMO for the oxidation of propane [41]; (vi) *T. butanivorans*, an alkane-oxidizing betaproteobacterium containing sHMO only [65]. Specific 1,7OD-dependent fluorescent staining of all tested cultures indicated efficient activity-based labeling of these alkane-oxidizing bacteria, regardless of enzyme type and phylogenetic affiliation (Fig. 2 and Supplementary Figs. S1 and S2). Still, the full range of hydrocarbon-degrading monooxygenases targeted by this method remains to be determined.

### Correlation of the activity-based staining with the bacterial growth stage

**Fig. 3:**
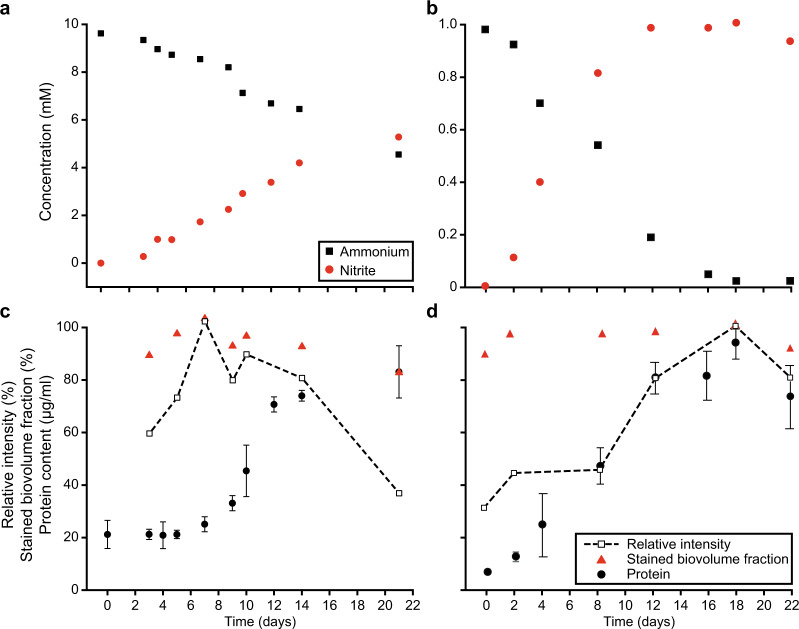
Correlation of the activity-based fluorescent labeling signal intensity and staining efficiency with the growth stage of ammonia-oxidizing bacteria. Ammonium consumption and nitrite production activity by **a** *Nitrosomonas europaea* and **b** *Nitrosospira multiformis* during the incubation period. Growth of **c** *N. europaea* and **d** *N. multiformis* as indicated by protein content, and its relation to the relative fluorescent signal intensity observed, as well as the biovolume fraction of the culture stained by the activity-based labeling protocol. Bacterial growth data are fitted to a Hill equation. Error bars represent standard deviation of technical triplicates of protein measurements.

Laboratory cultivation conditions poorly reflect the conditions microorganisms encounter in natural and engineered ecosystems. In these environments, fluctuations in substrate and nutrient availability rarely permit the continuous growth of microorganisms, including AOB [104]. To investigate the potential effect of the bacterial growth stage on the efficiency of the activity-based staining, *N. europaea* and *N. multiformis* pure cultures were cultivated in the presence of 10 or 1 mM ammonium, respectively, and ammonia-oxidizing activity and growth were monitored over 22 days. The cultures stoichiometrically oxidized ammonium to nitrite (Fig. 3a, b) and biomass samples taken at different time points were subjected to the activity-based labeling protocol. For *N. europaea*, the obtained fluorescent signal intensity reached its maximum at the start of the exponential growth phase (day 7) and decreased when the culture entered early stationary phase, but maintained a low signal intensity (35% compared to the maximum intensity) even in late stationary phase (Fig. 3c). Throughout all growth stages of the culture a high percentage (>90%) of cells was stained, with a small decrease (16%) in staining efficiency observed only in late stationary phase (Fig. 3c). In the case of *N. multiformis*, the activity-based staining-derived fluorescent signal reached its maximum intensity at the end of the exponential growth phase (day 18) and decreased by 30% once the culture reached its stationary phase (Fig. 3d). Also for this AOB a staining efficiency >86% was observed throughout all growth stages (Fig. 3d).

### Subcellular localization of active AMO and p/sMMO enzymes

**Fig. 4:**
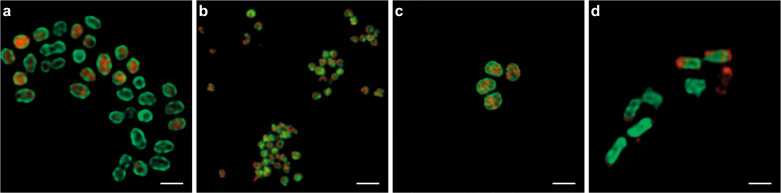
Subcellular localization of the active enzymes using activity-based fluorescent labeling signal and deconvolution confocal microscopy. Cells of **a** *Nitrosomonas europaea*, **b** *Nitrospira inopinata*, **c** *Methylotetracoccus oryzae*, and **d** *Methylocella tundrae* are stained with the fluorescent activity-based labeling protocol (shown in green) and specific FISH probes (shown in red): **a** AOB (Nso190, Neuo653, Nso1225), **b** *Nitrospira* (Ntspa662, Ntspa712), **c** Gammaproteobacteria (Gam42a), and **d** Alphaproteobacteria (Alf0001b, Alf0968). The scale bars correspond to 1 µm.

AMO and pMMO like all enzymes within the CuMMO protein family are known to be membrane-integral [42], with their active center on the periplasmic face of the cytoplasmic membrane or the ICM systems [105, 106]. In contrast, the sMMO is a soluble cytoplasmic enzyme [37]. To confirm these subcellular localizations, pure cultures of the canonical AOB *N. europaea*, the comammox bacterium *N. inopinata*, the type Ib methanotroph *M. oryzae* (containing only pMMO), and the methanotroph *M. tundrae* (containing only sMMO) were subjected to the activity-based staining protocol in combination with FISH and subsequent deconvolution fluorescence microscopy (Fig. 4). In *N. europaea*, the AMO label was observed mainly in a thick layer surrounding the cytoplasm (Fig. 4a), coinciding with the localization of the cytoplasmic membrane and the peripheral organization of the ICM system in *Nitrosomonas* [106–108]. In *N. inopinata* the small cell size (≤1 μm) precluded an unambiguous localization of the derived fluorescent signal, but the intense activity-based labeling indicated the presence of high amounts of active AMO enzymes along the cell membranes (Fig. 4b). In *M. oryzae* the ICMs are arranged as stacks of vesicular discs located in the central cytoplasm [105], which agrees with the observed strong pMMO fluorescent staining on apparently organized structures within the cytoplasm (Fig. 4c). As expected, in *M. tundrae* the sMMO labeling was distributed throughout the cytoplasm of the cell (Fig. 4d); however, there was only partly overlap with the 16S rRNA-derived signal, indicating that methane oxidation and translation occur in discrete locations within the cell.

**Fig. 5:**
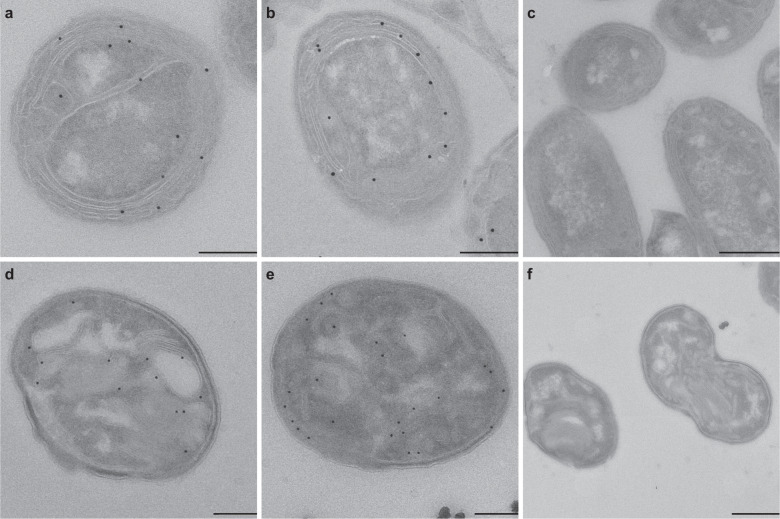
Immunogold activity-based labeling of active enzymes using transmission electron microscopy. Electron micrographs showing **a**–**c** *Nitrosomonas europaea* and **d**–**f** *Methylotetracoccus oryzae* cells pre-incubated (**a**, **b**, **d**, **e**) with and (**c**, **f**) without 1,7OD (control). Scale bars correspond to 0.5 μm.

In order to examine the localization of active AMO and pMMO enzymes at higher resolution, cells of *N. europaea* and *M. oryzae* were subjected to a modified version of the activity-based staining protocol. Here, the CuAAC reaction was used to biotinylate the AMO/pMMO enzymes within active bacterial cells, with subsequent immunogold labeling and transmission electron microscopy, thus providing high-resolution information on the subcellular localization of the complexes (Fig. 5). In *N. europaea*, 74% of the immunogold labeling indicated a membrane localization of the AMO complex, and the majority of these gold particles (49%) appeared to face the periplasmic space, with only 13% oriented toward the cytoplasm (Fig. 5a, b and Supplementary Table S3). In accordance to the proposed localization of the alkynylation site in the MOB *Methylococcus capsulatus* [52], 61% of gold labeling in *M. oryzae* depicted a membrane localization of the pMMO active center, with 71% of these membrane-associated labels on the periplasmic side of the membrane (Fig. 5d, e and Supplementary Table S3). In both cases, gold particles were seldomly found in the cytoplasm or outside the cell, indicating the high specificity of the labeling protocol (Fig. 5 and Supplementary Table S3).

### In situ activity-based fluorescent labeling of ammonia oxidizers in nitrifying enrichments

**Fig. 6:**
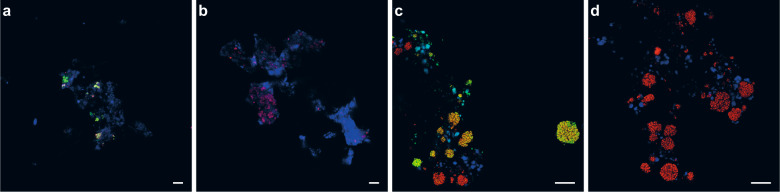
Activity-based fluorescent labeling of catalytically active ammonia-oxidizing bacteria present in complex microbial communities. Fluorescent micrographs showing **a**, **b** a culture containing *Ca*. N. nitrosa and *Ca*. N. nitrificans and **c**, **d** a nitrifying enrichment culture pre-incubated (**a**, **c**) with and (**b**, **d**) without 1,7OD (control). Cells are stained with the activity-based labeling protocol (shown in green) and FISH probes specific for *Nitrospira* (Ntspa662, Ntspa712; shown **a**, **b** in red and **c**, **d** in blue), **c**, **d** ammonia-oxidizing bacteria (Nso190, NEU653, Nso1225; in red), and **a**, **b** all bacteria (EUB338mix; in blue). Scale bars correspond to 10 µm.

The ability of the activity-based labeling protocol to stain ammonia oxidizers present in mixed microbial communities was assessed in different nitrifying enrichment cultures. We tested our newly developed protocol on the previously described enrichment culture of *Ca*. N. nitrosa and *Ca*. N. nitrificans [14], and a nitrifying enrichment containing canonical AOB, and both comammox and canonical *Nitrospira*, in addition to a range of putatively heterotrophic organisms. Furthermore, we demonstrated that a combination of the activity-based labeling protocol with 16S rRNA-targeted FISH allowed the simultaneous phylogenetic identification of the ammonia-oxidizing bacterial populations in all samples (Fig. 6).

The activity-based labeling protocol was able to stain both types of AOB present in these enrichment cultures. Fluorescent labeling of the AMO of comammox bacteria was observed both in the culture containing *Ca*. N. nitrosa and *Ca*. N. nitrificans (Fig. 6a), and in the nitrifying enrichment culture (Fig. 6c). In the latter, canonical betaproteobacterial AOB were also efficiently double-stained by the activity-based labeling protocol and FISH, while some *Nitrospira* were detected by FISH only, presumably corresponding to canonical *Nitrospira* present in this sample.

### Targeted metagenomics

In combination with FACS, our activity-based labeling protocol could be employed for targeted metagenomics of CuMMO and sHMO-containing bacteria present in complex environmental samples. To prove the feasibility of this approach, we tested the protocol on two samples of different complexity, a nitrifying enrichment culture and activated sludge from a full-scale WWTP.

**Fig. 7:**
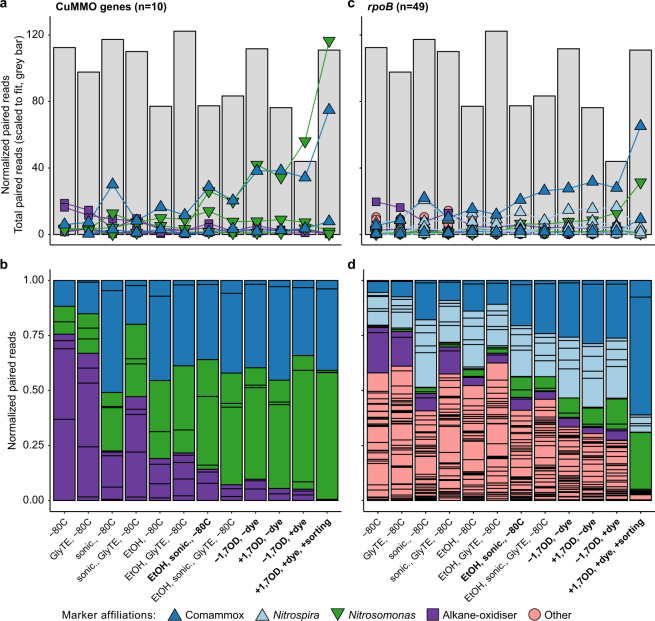
Targeted metagenomics of CuMMO-containing bacteria. Normalized read abundances of **a**, **b** CuMMO genes and **c**, **d** *rpoB* in the nitrifying enrichment culture of untreated biomass, controls, and sorted biomass for the activity-based labeling protocol (see Supplementary Table S2 for details). Gene abundances were calculated by normalizing paired read counts by gene length and million reads mapped to the co-assembly. The total number of reads mapped to the co-assembly are indicated as gray bars scaled to fit in the same range as the normalized abundances. Colors denote the taxonomic affiliation of the marker genes. The same genes for the taxa of interest (*Nitrosomonas*, comammox *Nitrospira*, and putative alkane oxidizers) are emphasized and connected with lines. **b**, **d** show the same data as (**a**, **c**) but as proportion of the total normalized read abundance for the respective gene in the metagenomic sample, with each individual colored box representing the relative abundance of a single gene from the co-assembly. The final treatment preceding the CuAAC reaction, the CuAAC controls for the influence of 1,7OD inactivation and dye addition, and the final sorted sample are indicated in bold.

Successive to the application of the activity-based labeling protocol and floc disruption using mild sonication, biomass of the nitrifying enrichment culture was used for FACS (Supplementary Fig. S3), with pooled collection of all positive sorting events. This fraction was subjected to DNA extraction and metagenomic sequencing, along with an untreated biomass sample and a range of controls to test for potential biases in the protocol (Supplementary Table S2). To demonstrate the efficacy of the labeling and sorting protocol in a manner unbiased by metagenomic binning approaches and different sequencing depths, we used metagenomic read mapping to assembled marker genes for specific functions, i.e., CuMMO genes, and microbial taxa, i.e., RNA polymerase subunit B (*rpoB*), to detect and differentiate all members of the microbial community. In total, ten CuMMO genes were identified in the co-assembled contigs retrieved from the nitrifying enrichment: three were highly similar to AMO genes of *Nitrosomonas* and two of comammox *Nitrospira* (>96% amino acid identity), while the remaining five were most similar to unresolved genes within proteobacterial lineages not known to oxidize ammonia (Supplementary Fig. S4). These latter genes clustered with putative pHMOs [42, 109] and became undetectable in the sorted sample. A total of 49 *rpoB* genes were recovered and analyzed, including two affiliated with *Nitrosomonas* (>90% amino acid identity), two with comammox *Nitrospira* (>97%), six with canonical *Nitrospira* (>97%), and two with *Candidatus* Macondimonas diazotrophica (>90%), a known hydrocarbon consumer that encodes a pHMO [110]. No *rpoB* genes affiliated with MOB or other lineages known to encode a CuMMO were apparent. For both marker genes belonging to the dominant ammonia oxidizers in the bioreactor, the normalized read abundances increased in the sorted samples compared to the unsorted samples: *Nitrosomonas* and comammox *Nitrospira amoA* increased 41- and 13-fold, and their *rpoB* 86 and 12-fold, respectively (Fig. 7). This increase in abundance was decoupled from the total number of reads per sample recruited to the co-assembly, indicating the changes were not due to variations in sequencing depth (Fig. 7). Finally, the abundance of non-ammonia-oxidizing community members’ *rpoB* genes decreased from 93.6% of the total normalized read abundance to 12.5%, indicating a strong de-enrichment of the microbial community members lacking AMO (Fig. 7). Therefore, the activity-based fluorescent labeling coupled to cell sorting successfully and specifically enriched the ammonia-oxidizing microorganisms present in the nitrifying enrichment bioreactor.

However, the activity-based labeling and sorting approach did reveal biases and limitations. First, there appeared to be enrichment of nitrifying bacteria due to treatment steps prior to sorting, particularly among the sonicated and ethanol-treated samples, but also by the CuACC reaction itself (Fig. 7). These effects may be due the tendency of nitrifying bacteria to grow as microcolonies within biomass aggregates, which may protect them from the effects of sonication, ethanol fixation, and the CuAAC reaction [92–94]. Second, the apparent most abundant ammonia oxidizer after activity-based labeling and cell sorting depended on the marker gene analyzed. Based on AMO gene abundance increases, a *Nitrosomonas*-like canonical AOB appeared to be more highly enriched, while this was a comammox *Nitrospira* according to *rpoB* abundance. The slopes of linear correlations (*r*^2^ > 0.95; Supplementary Fig. S5) of the most abundant CuMMO and corresponding *rpoB* genes support genomic evidence that this discrepancy may be an effect of gene copy number, as *Nitrosomonas* species typically encode three copies of the AMO gene but comammox *Nitrospira* only one [111]. As a side note, one putative bacterial hydrocarbon degrader-affiliated *rpoB* was strongly correlated (*r*^2^ > 0.9) to two different, similarly abundant putative pHMO genes across all samples, suggesting these microorganism may use distinct CuMMO enzymes able to activate a variety of alkanes (Supplementary Fig. S5); however, strong de-enrichment of this putative alkane oxidizer during sorting indicated that these pHMOs were not active under the incubation conditions of the enrichment culture (Fig. 7). Third, the larger increases in abundance of both *Nitrosomonas* marker genes compared to comammox *Nitrospira* suggest that the technique may slightly favor *Nitrosomonas*-like AOB. Fourth, some AMO genes and *rpoB* affiliated with different ammonia oxidizers were either poorly enriched or even de-enriched, which makes it tempting to speculate that the physiology of these seemingly related populations differ in vivo to the extent that some do not express AMO (Fig. 7). While it is important to acknowledge and account for these caveats, metagenomic recovery and abundance estimations clearly showed effective enrichment of ammonia-oxidizing microorganisms by the activity-based labeling protocol coupled to FACS.

**Fig. 8:**
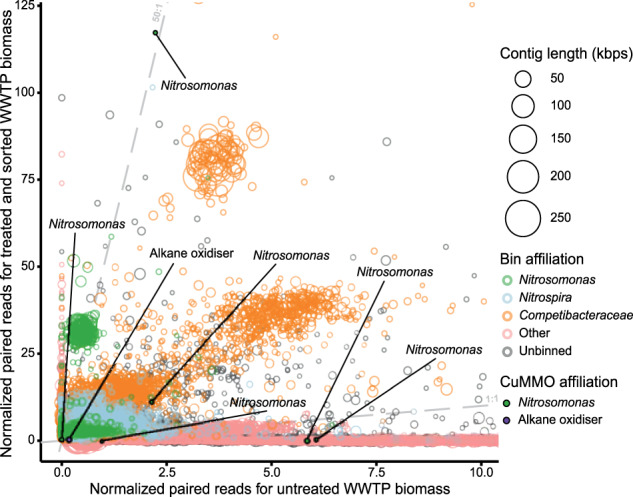
Differential coverage plot showing the abundance of contigs in the municipal WWTP untreated activated sludge sample in comparison to the activity-based labeled and sorted sample. Contig abundances were calculated by normalizing paired read counts by gene length and million reads mapped to the assembly. Each circle represents an assembled contig ≥1500 bps, indicated by circle size. Contigs assigned to the automated bins of taxa of interest are highlighted using specific colors. Contigs with genes encoding members of the CuMMO protein family are emphasized with fill circles and text. For added clarity, dashed lines were drawn with a slope of 1 and 50 to guide estimations of the differences in normalized abundances between the samples. The plot is zoomed in on the region containing >80% of all data, the full version is provided as Supplementary Fig. S6.

When our activity-based labeling protocol was applied to activated sludge sampled at a municipal WWTP, bacterial ammonia-oxidizing populations were also successfully enriched. Co-assembly of the untreated sludge biomass, controls, and sorted samples yielded two *Nitrosomonas* bins and two *Nitrospira* bins (Supplementary Table S4). The two *Nitrosomonas* bins increased 8- and 53-fold in mean normalized read abundance compared to the untreated biomass, while the *Nitrospira* bins increased 4-fold (Fig. 8 and Supplementary Fig. S6). These four bins accounted for 1.1% of the normalized reads in the original sample and less than 20% of their contigs were detected (covered bases >50%), but after activity-based labeling and sorting these bins constituted 11.5% of the normalized reads, suggesting that sorting was able to aid in the recovery of otherwise inaccessible genomes of AOB. Though not automatically binned, four partial and two full-length *Nitrosomonas*-affiliated AMO genes were assembled, as well as one putative pHMO gene affiliated with Proteobacteria (Supplementary Fig. S4). No comammox *Nitrospira*-related AMO gene was detected in the assembly, indicating that the enriched *Nitrospira* bins belonged to canonical nitrite-oxidizing species that were sorted together with their ammonia-oxidizing partners due to their close physical interaction [112]. The two full-length *Nitrosomonas* AMO genes showed similar patterns as those in the nitrifying enrichment bioreactor because they were approximately 3.8-fold more abundant in the sorted metagenome than the likely bin. Assembly and binning of the sorted sample alone led to the recovery of a high-quality *Nitrosomonas* MAG (>99% complete, <1% contaminated), enabling the assignment of one of the *Nitrosomonas* AMO genes to this MAG (Supplementary Fig. S4). Contrastingly, the low abundance and lack of matching abundance patterns with any bin prevented assignment of the putative pHMO. Thus, despite the challenges of assembling and binning closely related strains of ammonia oxidizers, the ABPP-based protocol in combination with targeted cell sorting was clearly capable of enriching low-abundant ammonia oxidizers from complex activated sludge samples.

Surprisingly, over 72% normalized reads obtained for the sorted activated sludge sample were recruited to bins affiliated with putative glycogen-accumulating organisms (GAOs) of the family *Competibacteraceae* (Supplementary Table S4). In addition, all eight automated bins increased in mean normalized read abundance approximately 4.7- to 18.5-fold, suggesting that they were also enriched by the activity-based labeling and sorting. Their high abundance in the sorted sample enabled the recovery of four medium-quality MAGs (61–96% complete, <10% contaminated) from an assembly of the sorted sample alone, which represent three distinct genera (Supplementary Table S5). No genes encoding known members of the CuMMO or sHMO protein families were detected in these bins or MAGs, suggesting that the apparent labeling (Supplementary Fig. S7) and enrichment by sorting was due to other factors. Instead, all but one of the *Competibacteraceae* MAGs encoded nitrogenases, which is an enzyme known to interact with alkynes and may explain the unexpected enrichment [113].

To test for potential labeling of the nitrogenase complex, we incubated pure cultures of the diazotroph *Krypidia spormannii* FAVT5 [66] and the methanotroph *M. oryzae* [64] under nitrogen-fixing conditions. Subsequently, both cultures were subjected to the activity-based labeling protocol. As *M. oryzae* contains a pMMO, staining of these cells was observed, but compared to a culture grown in the presence of nitrate as N-source in the medium, the staining intensity was slightly reduced (Supplementary Fig. S2). For *K. spormannii* FAVT5, the amount of signal observed after the CuAAC reaction was just above background, corresponding to approximately 12% and 25% of the signal obtained from active *N. europaea* and *M. tundrae* cultures, respectively (Supplementary Fig. S2). Together, these results indicate that the presence of an active nitrogenase does minimally contribute to the activity-based labeling, making the reason for the observed staining of the *Competibacteraceae*-affiliated MAGs unclear. It thus remains to be discovered which enzymes apparently are able to react with 1,7OD and what their natural function is in the metabolism of these *Competibacteraceae* species.

## Discussion

ABPP-based protocols have been successfully employed for the study of many microbial protein families, such as proteases, kinases, hydrolases, and glycosidases [20, 29]. However to date, they have rarely been applied for the in situ labeling of whole cells within microbial communities, due to redundant, poorly resolved, or unclearly connected taxa or functions [114]. In situ use of ABPP-based protocols poses major challenges for employing the CuAAC reaction, for example probe uptake, and labeling specificity and efficiency [115]. The activity-based labeling protocol developed in this study overcomes these challenges and allows the fluorescent visualization of specific catalytically active enzymes in situ. The protocol makes use of the diyne 1,7OD, which serves as a bifunctional enzyme probe for the specific detection of CuMMO and sHMO enzymes in diverse ammonia- and alkane-oxidizing bacteria. The feasibility of this approach was previously demonstrated in vitro for the AMO of canonical AOB [31], but had not been tested for labeling whole cells, phylogenetically distinct ammonia-oxidizing microorganisms, or evolutionarily or functionally related enzymes. Generally, *n*-terminal and sub-terminal alkynes are known mechanism-based inactivators of AMO [53, 54] and p/sMMO enzymes [51, 56]. Here, we revealed that 1,7OD also inhibits ammonia oxidation in comammox *Nitrospira* (Fig. 1) as efficiently as was reported for canonical AOB [31]. Furthermore, strong labeling of different methane- and other alkane-oxidizing bacteria harboring diverse pHMO or sHMO enzymes (Fig. 2) indicated that methanotrophs and alkane-oxidizing bacteria can also be inhibited by this diyne.

Application of the in situ activity-based protocol on pure and enrichment cultures of different canonical AOB, comammox *Nitrospira*, and alkane oxidizers resulted in efficient, mechanism-based fluorescent labeling (Fig. 2 and Supplementary Fig. S2). This directly conveyed information on the functional potential of the detected microorganisms, in contrast to conventional in situ detection methods like FISH. Still, in most natural and engineered systems, microorganisms rarely encounter optimal growth conditions and might co-exist in heterogeneous growth stages. Previous studies have identified preservation of basal activity levels of ammonia- and methane-oxidizing microorganisms as a general adaptation to cope with alternating conditions and fluctuating substrate supply [104, 116], which therefore maintain a basal CuMMO content. Quantification of the activity-based fluorescent labeling signals in *N. europaea* and *N. multiformis* pure cultures showed strong correlations between signal intensity and growth state (Fig. 3). As expected, the strongest signals were observed during the exponential growth phase, corresponding to a high AMO content of the cells. Lower but detectable labeling was still observed even in the late stationary phase of the cultures, demonstrating that basal AMO contents are also maintained in non-replicating cells. Thus, although the intensity of the activity-based labeling might vary, the approach is suitable for the detection of ammonia- and methane-oxidizing cells in different growth stages. However, exposure to their specific substrate may be required to stimulate expression of the target enzymes by metabolically versatile microorganisms for this activity-based labeling protocol, as indicated by the lack of labeling of some *Nitrosomonas* species and the pHMO-containing putative alkane oxidizers detected in the nitrifying enrichment culture (Fig. 7).

Unfortunately, it was not possible to label the archaeal AMO using 1,7OD as bifunctional enzyme probe (Supplementary Fig. S2i), consistent with the low sensitivity of AOA to longer-chain length (>C_5_) alkynes and their reversible inhibition by 1-octyne [54]. Nevertheless, these microorganisms are sensitive to inactivation by *n*-alkynes with short carbon backbones (≤C_5_) [31], and thus substituting 1,7OD with an alkyne with a smaller chain length might permit labeling of archaeal AMO. However, the fact that diynes smaller than 1,5-hexadiyne are extremely reactive and difficult to obtain makes modification of this method for the detection of AOA challenging.

Using high-resolution deconvolution laser scanning microscopy as well as electron microscopy, the activity-based labeling protocol permitted visualization of the subcellular localization of the AMO and p/sMMO enzymes in AOB and MOB (Figs. 4 and 5). In accordance with previous studies [63, 107, 108], the AMO and pMMO-derived fluorescent signals were mostly localized along the cytoplasmic membrane and on the ICM stacks in *N. europaea* and *M. oryzae*, respectively, whereas a cytoplasmic localization of the sMMO-derived signal was observed for *M. tundrae*. Furthermore, the immunogold labeling data obtained in this study suggested that the alkynylation sites for both the AMO and pMMO complexes were found on the periplasmic face of the ICMs. This congruence with expected enzyme localizations further verified the suitability of the activity-based protocol for the specific labeling of CuMMO enzymes, which opens up possibilities for studying differences in enzyme distribution along intracellular structures in AOB and alkane-oxidizing microorganisms with divergent cell morphologies. Although in theory direct tagging of the CuMMO using azide-functionalized gold nanoparticles could be employed in future enzyme localization studies, the biotinylation of these enzymes via the activity-based protocol as tested here can also provide a new methodology for the profiling or affinity purification to study various CuMMO and sHMO enzymes.

Generally, nitrifiers and methane oxidizers are notoriously difficult to cultivate due to their slow growth rates and susceptibility to contamination. Besides classical cultivation attempts, the identification of novel ammonia and methane oxidizers to date was achieved using cultivation-independent molecular techniques [117–119] and metagenomic sequencing [2, 13–17, 120–123], which has been invaluable for providing insights into the distribution and ecological importance of these functional groups. Still, due to the complexity of natural ecosystems, metagenomic sequencing frequently fails to recover sufficient data to reconstruct genomes of low-abundance organisms, and linking novel sequence types detected in metagenomic or PCR-derived datasets to a specific phylotype often is a highly challenging and time-consuming task. In addition, PCR primers will mostly fail to amplify novel and divergent sequences, thereby hindering the detection of species with novel metabolic capabilities, as was showcased by the recent discovery of complete nitrification within members of the genus *Nitrospira* [13, 14]. Thus, there is a pressing need for simple detection methods that do not require a priori knowledge on the identity of microorganism. The here-developed activity-based labeling protocol can be combined with FISH to directly link the function of a population to its identity in situ, which could help to identify novel microorganisms capable of utilizing ammonia, methane, or other short-chain alkanes. Along these lines, while *amoA*-targeting PCR-based methods by now have successfully been employed to detect the presence and abundance of comammox *Nitrospira* in complex microbial communities or environmental samples [117, 124, 125], it remains challenging to phylogenetically differentiate canonical and comammox *Nitrospira*. In combination with FISH, the activity-based staining method described here overcomes this limitation and enables an reliable differentiation of comammox and canonical *Nitrospira* in mixed microbial communities (Fig. 6).

Furthermore, we combined the activity-based labeling protocol with cell sorting and subsequent metagenomic sequencing. Application of this protocol on a nitrifying bioreactor enrichment culture successfully enriched the *Nitrosomonas*-like betaproteobacterial AOB and comammox *Nitrospira* (Fig. 7). While the physical and chemical treatments of the biomass during the activity-based protocol introduced biases resulting in a slight unspecific enrichment of target populations also in the controls, sorting was highly specific for these ammonia-oxidizing microorganisms (Fig. 7). As discussed above, the observed unspecific enrichment may be due to the tendency of these bacteria to physically associate in microcolonies [126, 127]. Regardless, the activity-based fluorescent labeling coupled to targeted cell sorting was sufficient for strong enrichment of multiple AOB from a mixed microbial community. When applied to activated sludge from a full-scale municipal WWTP, the activity-based labeling protocol with subsequent cell sorting resulted in strong enrichment of *Nitrosomonas*-like AOB (Fig. 8), which initially were at low abundance and constituted <1% of normalized reads in this sample. The activity-based labeling followed by cell sorting enriched them over 50-fold and even enabled recovery of a high-quality *Nitrosomonas* MAG. With deeper sequencing, or for samples with sufficient biomass with additional long-read sequencing, it will likely even be possible to recover circular genomes using this targeted metagenomics approach.

Surprisingly, several MAGs belonging to members of the *Competibacteraceae* family were enriched from the activated sludge sample after activity-based labeling and sorting. Described *Competibacteraceae* species are GAOs, which frequently are encountered in enhanced biological phosphorus removal systems. They compete with phosphate-accumulating organisms for resources under the cyclic anaerobic feast—aerobic famine regime and thus reduce the phosphate removal efficiency of these wastewater treatment systems [128]. Their primary metabolism includes synthesis and storage of glycogen and polyhydroxyalkanoates, but they are also capable of fermentation, denitrification, and nitrogen fixation [129]. However, these bacteria have never been implicated in the nitrification process or hydrocarbon degradation, and the MAGs recovered here lacked genes that are known to react with 1,7OD during the activity-based labeling approach. Therefore, the reason for their enrichment (Fig. 8 and Supplementary Fig. S7) through the activity-based labeling protocol remains unclear and requires future research.

In conclusion, we present a novel activity-based protocol that enables the efficient in situ labeling of CuMMO and sHMO-encoding bacteria, can be used to better link function and identity, and facilitates the targeted retrieval of high-quality and metabolically constrained genomes for microbes of low abundance. Although optimization of the protocol for difficult sample types that exhibit high background fluorescence will be necessary, the developed activity-based labeling protocol is a significant milestone for integrating phylogenetic and physiological information that can be adapted and applied to complex microbial communities. Coupling this novel method with other common techniques can help reveal the functional differences in biomass aggregates of phylogenetically indistinguishable lineages, as well as enable the recovery of new genomic representatives critical for engineered processes that account for only a small portion of the microbial community. Therefore, the future application of this activity-based labeling protocol will contribute greatly to an improved understanding of the microorganisms involved in important carbon and nitrogen cycle processes within a variety of ecosystems.

## Supplementary information

Supplementary Information

Supplementary Table S4

Supplementary Table S5

## Data Availability

Sequencing data obtained in this study have been deposited in the National Center for Biotechnology Information (NCBI) database under BioProject accession numbers PRJNA691748 (nitrifying enrichment culture) and PRJNA691751 (activated sludge sample).
