# Supplementary material for: Universal activity-based labeling method for ammonia- and alkane-oxidizing bacteria
Source: ISME J. 2021 Nov 6;16(4):958–71. doi: 10.1038/s41396-021-01144-0 (PMC8941013; doi:10.1038/s41396-021-01144-0)
Supplement: Supplementary file 1 — Supplementary Information [file 41396_2021_1144_MOESM1_ESM.pdf]

Supplemental Information for:

Universal activity-based labelling method for ammonia- and alkane-oxidizing bacteria

Dimitra Sakoula[1#*], Garrett Smith[1], Jeroen Frank[1,2], Rob. J. Mesman[1], Linnea F.M. Kop[1], Pieter Blom[1], Mike S.M. Jetten[1,2], Maartje A.H.J. van Kessel[1], Sebastian Lücker[1*]

[1]Department of Microbiology, IWWR, Radboud University, Heyendaalseweg 135, 6525 AJ Nijmegen, the Netherlands

[2]Soehngen Institute of Anaerobic Microbiology, Radboud University, Heyendaalseweg 135, 6525 AJ Nijmegen, the Netherlands

[#]Present address: Division of Microbial Ecology, Center for Microbiology and Environmental Systems Science, University of Vienna, Althanstraße 14, 1090, Vienna, Austria

***Corresponding authors:**

Dimitra Sakoula, Division of Microbial Ecology, Center for Microbiology and Environmental Systems Science, University of Vienna, Althanstraße 14, 1090, Vienna, Austria; phone: +43 1 4277 91201, mail: dimitra.sakoula@univie.ac.at

Sebastian Lücker, Department of Microbiology, IWWR, Radboud University, Heyendaalseweg 135, 6525 AJ Nijmegen, the Netherlands; phone: +31 24 3652618, mail: s.luecker@science.ru.nl

Supplemental Figures and Tables:

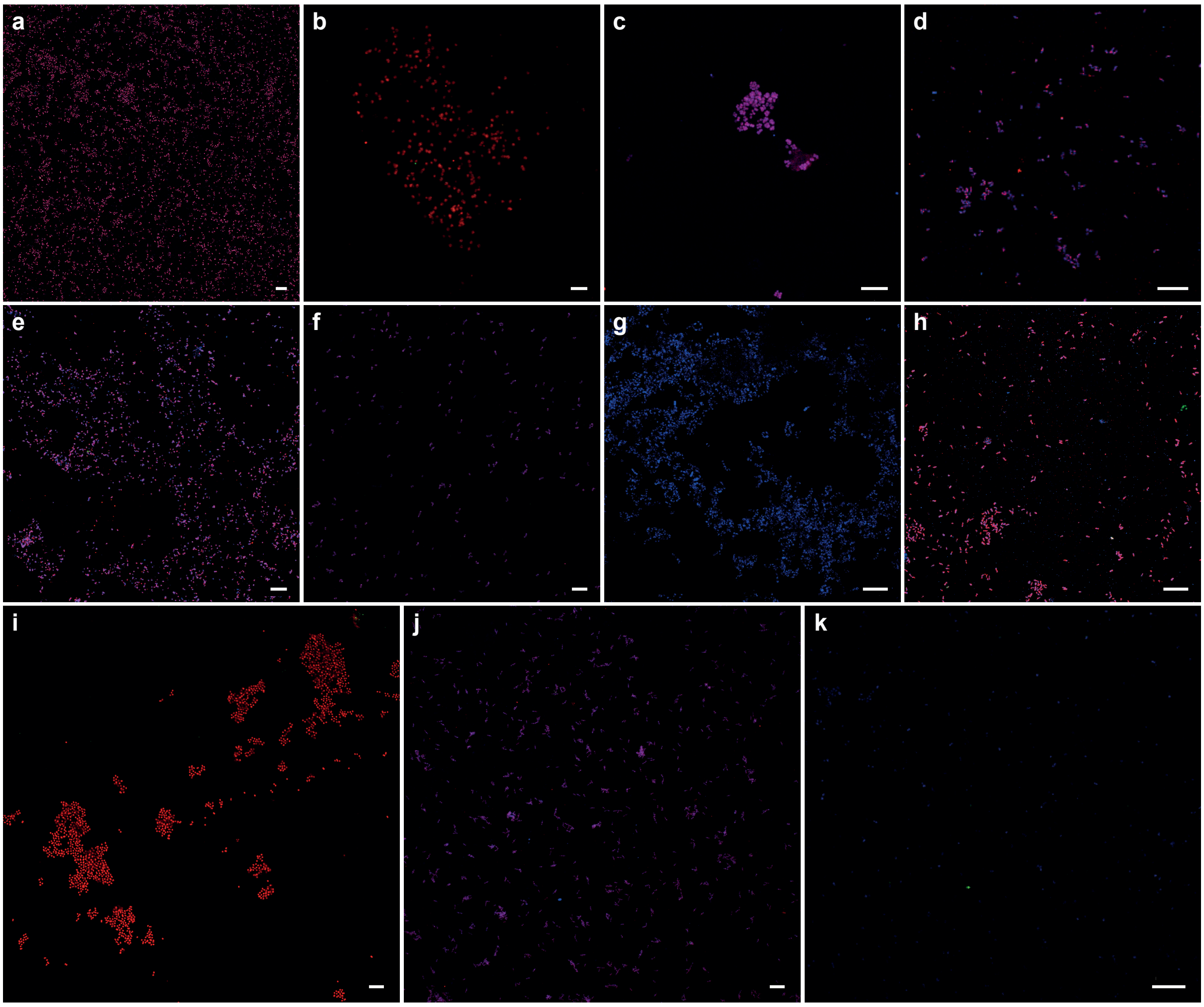

Figure S1. Control incubations of catalytically active ammonia-, methane- and other alkane-oxidizing microorganisms. Fluorescent micrographs of cells pre-incubated (**a-h**) without 1,7OD of (**a**) *Nitrosomonas europaea*, (**b**) *Nitrospira inopinata*, (**c**) *Methylotetracoccus oryzae*, (**d**) *Methylosinus sporium* M29, (**e**) *Methylacidiphilum fumariolicum* SolV, (**f**) *Methylocella tundrae*, (**g**) *Rhodococcus* sp. strain ZPP, (**h**) *Thauera butanivorans*, and (**i-k**) with 1,7OD of (**i**) *Nitrosocosmicus franklandus*, (**j**) *Nitrospira moscoviensis*, and (**k**) *Escherichia coli*. Cells were subjected to the CuAAC reaction in the presence of Fluor488-azide (shown in green) and hybridized with FISH probes specific for (**a**) AOB (Nso190, Neu653, Nso1225; red), (**b, i**) *Nitrospira* (Ntspa662, Ntspa712; red), (**c, f**) Gammaproteobacteria (Gam42a; red), (**d**) Alphaproteobacteria (Alf0001b, Alf0968; red), (**e**) Verrucomicrobia (EUB338III; red), (**h**) Betaproteobacteria (Bet42a; red), (**a-f, h, i**) all bacteria (EUB338mix; blue) and (**j**) all archaea (ARCH915; red). Cells in (**g, k**) were counterstained with DAPI (blue). Note that the green spot in (**k**) was an autofluorescent particle on the slide surface. Scale bars correspond to 10 µm.

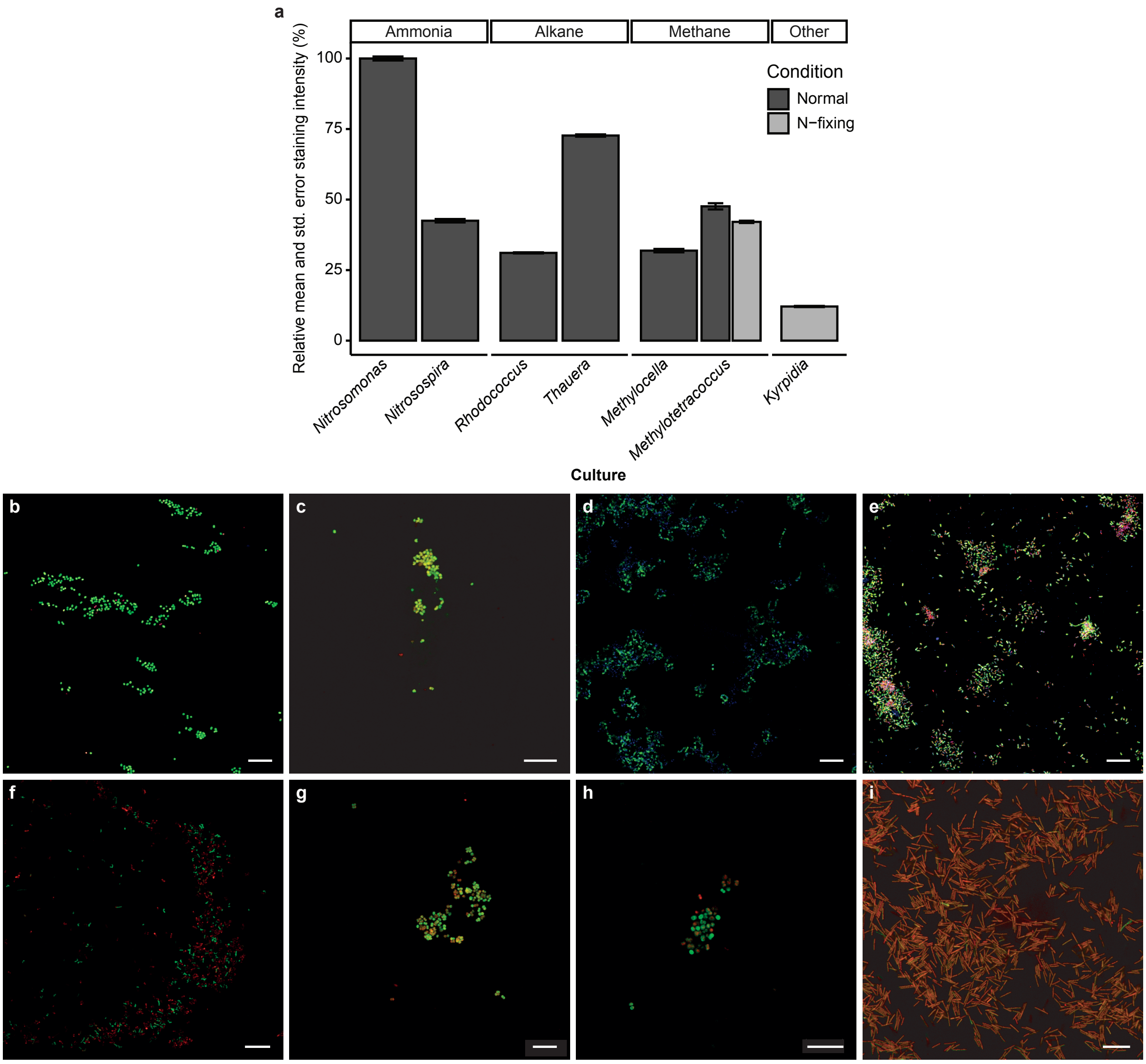

Figure S2. Activity-based staining of representative CuMMO and sHMO-containing bacteria. (**a**) Observed relative signal intensity of the activity-based fluorescent labelling and (**b-i**) fluorescent micrographs of (**b, c**) ammonia-oxidizing (**b**) *Nitrosomonas europaea* and (**c**) *Nitrosospira multiformis*, (**d**) propane-oxidizing *Rhodococcus* sp. strain ZPP, (**e**) butane-oxidizing *Thauera butanivorans*, (**f-h**) methane-oxidizing (**f**) *Methylocella tundrae* and (**g, h**) *Methylotetracoccus oryzae* (**g**) with N-source supplied and (**h**) under N-fixing conditions), and (**i**) heterotrophic *Kyrpidia spormannii* FAVT5 under N-fixing conditions. Cells were stained with the activity-based labelling protocol (shown in green) and FISH probes specific for (**b, c**) AOB (Nso190, Neu653, Nso1225; red), (**e**) Betaproteobacteria (Bet42a; red, and EUB338-mix, blue), (**f-h**) Gammaproteobacteria (Gam42a; red), and (**i**) all bacteria (EUB338mix; red). Cells in (**d**) were counterstained with DAPI (blue). Scale bars correspond to 10 µm.

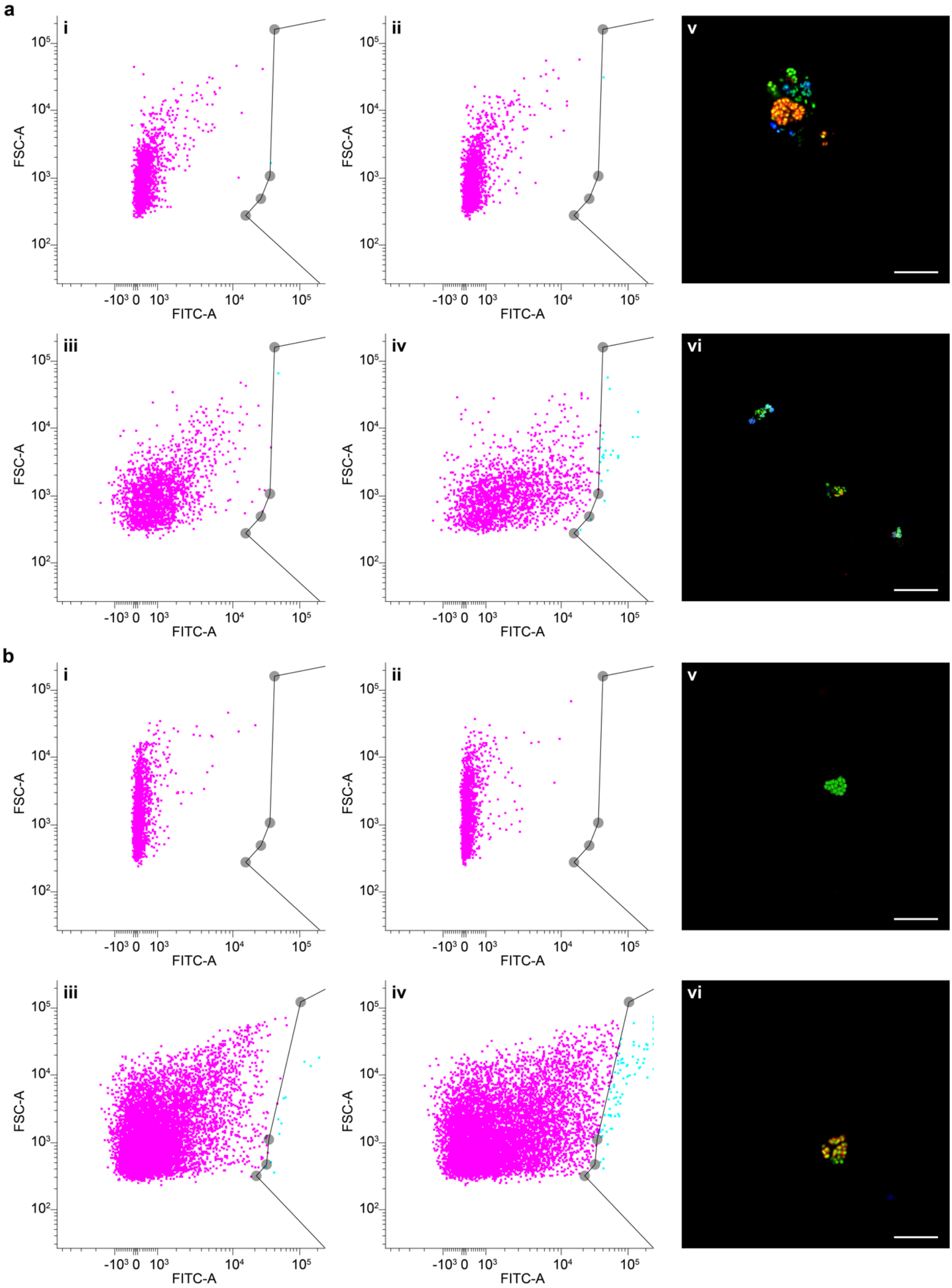

Figure S3. Gating of the activity-based labelled fluorescent subpopulation of (**a**) the nitrifying enrichment and (**b**) activated sludge. (i) Biomass incubated without 1,7OD and subjected to a CuAAC reaction without Fluor488-azide, (ii) biomass incubated with 1,7OD and subjected to a CuAAC reaction without Fluor488-azide, (iii) biomass

incubated without 1,7OD and subjected to a CuAAC reaction, (iv) biomass incubated with 1,7OD and subjected to a CuAAC reaction, (v, vi) representative FISH micrographs of the sorted biomass showing the activity-based labelling protocol (green), and specific FISH probes for AOB (Nso190, Nso1225, NEU653; red) and *Nitrospira* (Ntspa662, Ntspa712; blue). Scale bars correspond to 10 µm.

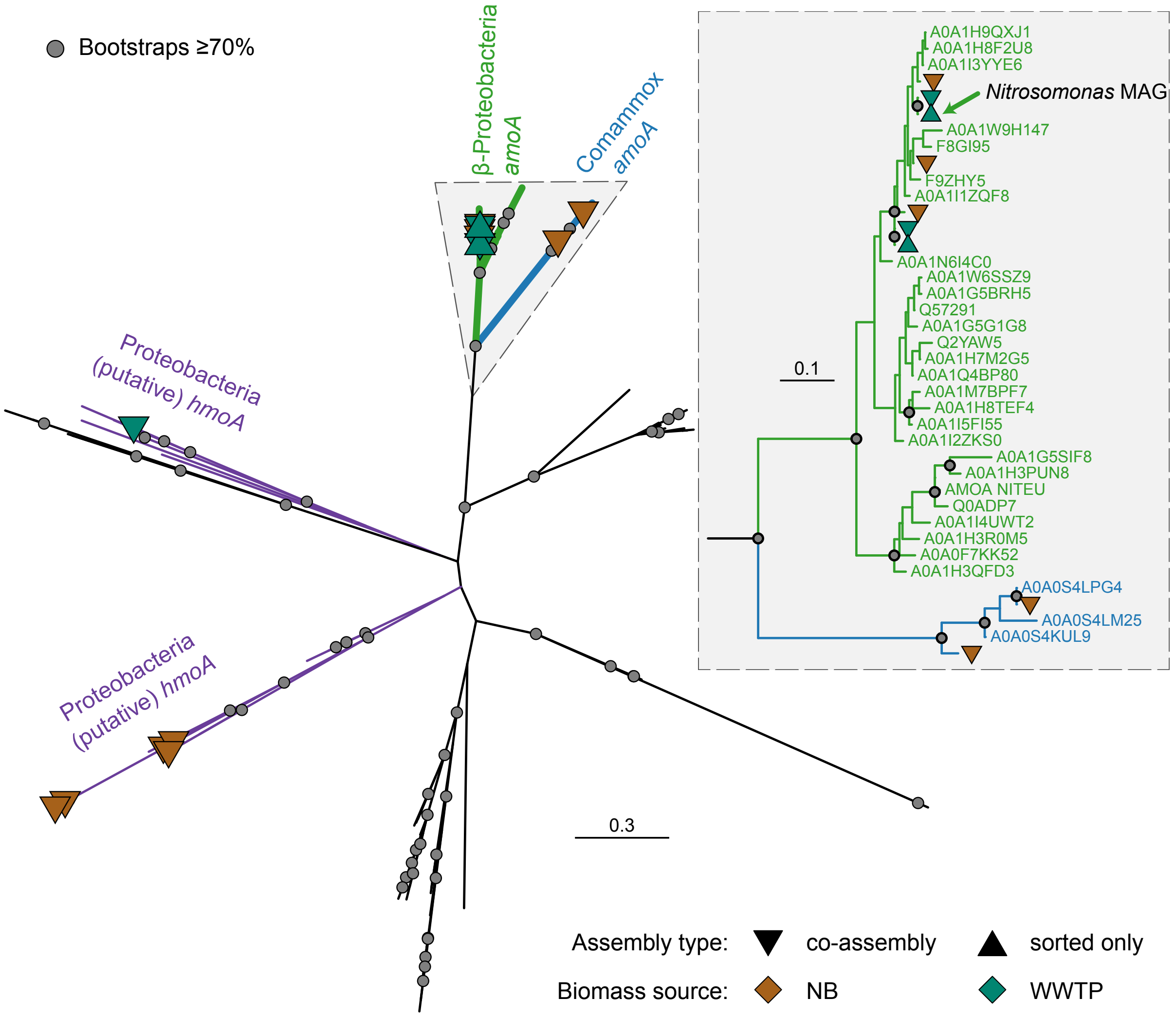

Figure S4. Unrooted phylogenetic reconstruction of full-length CuMMO gene references (Pfam PF02461) and genes recovered from three metagenomic assemblies. Circles indicate nodes with statistical support, branch colors highlight clades of interest, and triangles denote the assembled genes while reference clades are unlabeled. The inset shows the outlined region of the tree to clarify the topology of this dense region, with the accession numbers of the reference sequences displayed. *amoA*, ammonia monooxygenase subunit A; *hmoA*, alkane monooxygenase subunit A; NB, nitrifying bioreactor; WWTP, wastewater treatment plant. The scale bar represents 0.3 amino acid substitutions per alignment position.

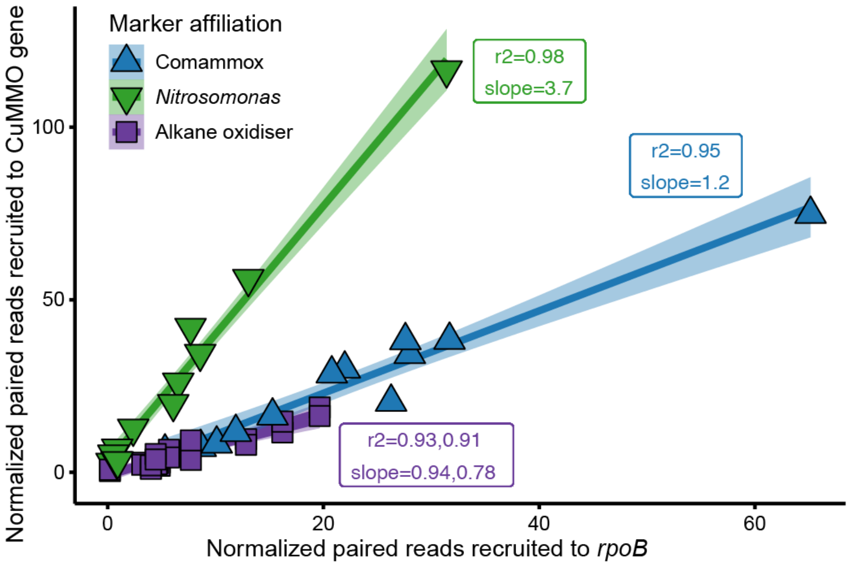

Figure S5. Linear correlations of the CuMMO gene and *rpoB* normalized read abundances of the dominant ammonia- and putative alkane-oxidizing bacteria in the nitrifying enrichment culture. Each symbol represents the Reads Per Kilobase gene length per Million mapped reads (RPKM) of a gene in one metagenomic sample, colored by taxonomic affiliation to match Figure 7. The linear relationship and the 95% confidence interval are shown as a line and background of matching color and are labelled with the coefficient and the slope of the line in matching color.

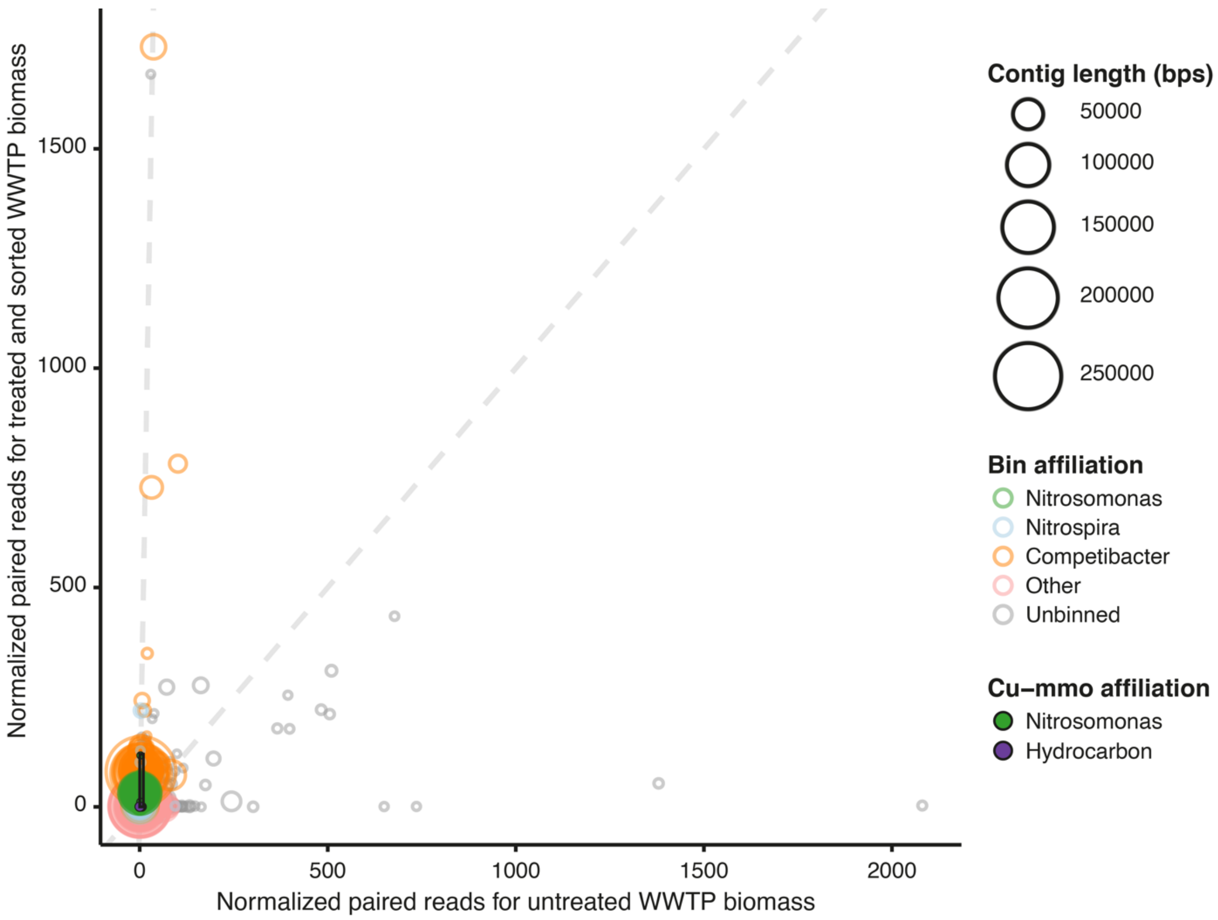

Figure S6. Differential coverage plot showing the abundance of contigs in the municipal WWTP untreated activated sludge sample in comparison to the activity-based labelled and sorted sample. Contig abundances were calculated by normalizing paired read counts by gene length and million reads mapped to the assembly. Each circle represents an assembled contig ≥1500 bps, indicated by circle size. Contigs assigned to the automated bins of taxa of interest are highlighted using specific colors. Contigs with genes encoding members of the CuMMO protein family are emphasized with fill circles and text. For added clarity, dashed lines were drawn with a slope of 1 and 50 to guide estimations of the differences in normalized abundances between the samples. The black rectangle indicates the region that is shown in Figure 8.

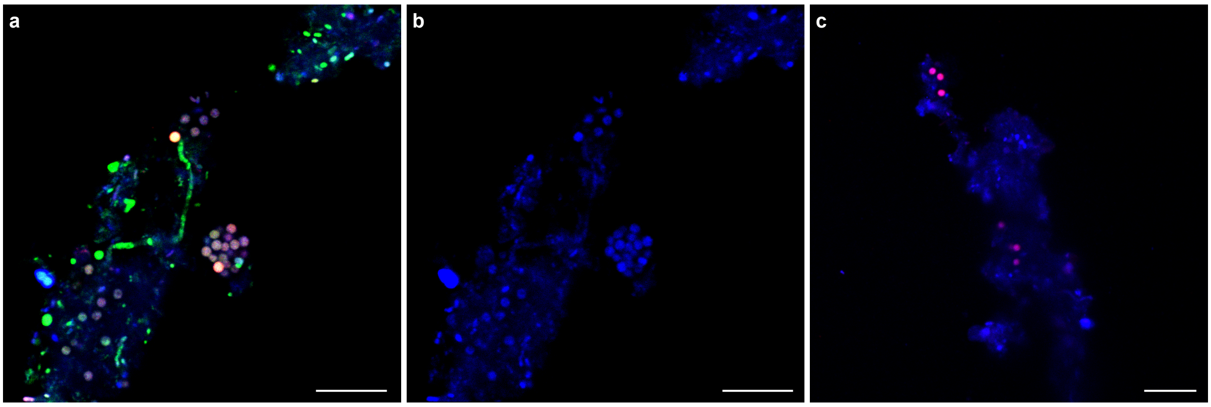

Figure S7. ABPP-based fluorescent labelling of (**a, b**) 1,7OD pre-incubated and (**c**) 1,7OD untreated (control) *Competibacteraceae* cells present in activated sludge from a municipal WWTP. Cells were stained with the activity-based labelling protocol (shown in green) and FISH probes targeting *Competibacteraceae* spp. (GAOQ431, GAOQ989; red) and all bacteria (EUBmix; blue). Panel (**b**) shows only the EUBmix signal of the region recorded in (**a**). Note that most cells labelled by the activity-based labelling protocol in green but not by the GAOQ431 and GAOQ989 FISH probes do show a weak EUBmix signal. As the employed probes do not target all *Competibacteraceae* MAGs obtained after sorting from this WWTP sample, these cells likely correspond to those species, which also is corroborated by the absence of additional bacterial species in the sorted sample. Scale bars correspond to 10 µm.

Table S1 | Specifications of the FISH probes used in this study

Probe	Target	% FA	Sequence (5'→3')	Reference[a]
Ntspa662	*Nitrospira* genus	35	GGA ATT CCG CGC TCC TCT	(1)
cNtspa662	Competitor to Ntspa662	-	GGA ATT CCG CTC TCC TCT	(1)
Ntspa712	*Nitrospira* phylum	35	CGC CTT CGC CAC CGG CCT TCC	(1)
cNtspa712	Competitor to Ntspa712	-	CGC CTT CGC CAC CGG TGT TCC	(1)
Bet42a	Betaproteobacteria	35	GCC TTC CCA CTT CGT TT	(2)
Gam42a	Gammaproteobacteria	40	GCC TTC CCA CAT CGT TT	(2)
Nso190	Betaproteobacterial AOB	45	CGA TCC CCT GCT TTT CTC C	(3)
Nso1225	Betaproteobacterial AOB	35	CGC CAT TGT ATT ACG TGT GA	(4)
NEU653	*Nitrosomonas* spp.	35	GCT GCC ACC CGT AGG TGT	(3)
cNEU653	Competitor to NEU653	-	TTC CAT CCC CCT CTG CCG	(3)
GAOQ431	*Competibacteraceae*	35	TCC CCG CCT AAA GGG CTT	(5)
GAOQ989	*Competibacteraceae*	35	TTC CCC GGA TGT CAA GGC	(5)
EUB338	Most Bacteria	0-80	GCT GCC TCC CGT AGG AGT	(6)
EUB338II	*Planctomycetales*	0-80	GCA GCC ACC CGT AGG TGT	(7)
EUB338III	*Verrucomicrobiales*	0-80	GCT GCC TCC CGT AGG AGT	(7)
ARCH915	Archaea	20-60	GTG CTC CCC CGC CAA TTC CT	(6)

[a] **References:**

1. Daims H, Nielsen JL, Nielsen PH, Schleifer KH, Wagner M. *In situ* characterization of *Nitrospira*-like nitrite-oxidizing bacteria active in wastewater treatment plants. Applied and Environmental Microbiology. 2001;67(11):5273-84.
2. Manz W, Amann R, Ludwig W, Wagner M, Schleifer K-H. Phylogenetic oligodeoxynucleotide probes for the major subclasses of Proteobacteria: Problems and solutions. Systematic and Applied Microbiology. 1992;15(4):593-600.
3. Wagner M, Rath G, Amann R, Koops H-P, Schleifer K-H. *In situ* Identification of ammonia-oxidizing bacteria. Systematic and Applied Microbiology. 1995;18(2):251-64.
4. Mobarry BK, Wagner M, Urbain V, Rittmann BE, Stahl DA. Phylogenetic probes for analyzing abundance and spatial organization of nitrifying bacteria. Applied and Environmental Microbiology. 1996;62(6):2156-62.
5. Crocetti GR, Banfield JF, Keller J, Bond PL, Blackall LL. Glycogen-accumulating organisms in laboratory-scale and full-scale wastewater treatment processes. Microbiology. 2002;148(11):3353-64.
6. Amann RI, Krumholz L, Stahl DA. Fluorescent-oligonucleotide probing of whole cells for determinative, phylogenetic, and environmental studies in microbiology. Journal of Bacteriology. 1990;172(2):762-70.
7. Daims H, Brühl A, Amann R, Schleifer K-H, Wagner M. The Domain-specific Probe EUB338 is insufficient for the detection of all bacteria: Development and evaluation of a more comprehensive probe set. Systematic and Applied Microbiology. 1999;22(3):434-44.

Table S2 | Detailed description of the controls included into the targeted metagenomics approach to determine the potential biases introduced by the different treatment steps of the activity-based protocol

Sample name[a]	Storage at -80°C	GlyTE buffer	Sonication	EtOH fixation	1,7OD addition	CuACC reaction	Azide-Fluor 488 addition	FACS
**-80°C (untreated)**	■							
GlyTE, -80°C	■	■						
Sonic, -80°C	■		■					
Sonic, GlyTE, -80°C	■	■	■					
EtOH, -80°C	■			■				
EtOH, GlyTE, -80°C	■	■		■				
EtOH, sonic, -80°C	■		■	■				
EtOH, sonic, GlyTE, -80°C	■	■	■	■				
**-1,7OD, -dye**	■	■	■	■		■		
**+1,7OD, -dye**	■	■	■	■	■	■		
**-1,7OD, +dye**	■	■	■	■		■	■	
**+1,7OD, +dye, sorting**	■	■	■	■	■	■	■	■

[a] Treatment controls in bold were included for both environmental samples, all others only for the nitrifying enrichment culture.

Table S3 | Subcellular localization of the immuno-gold labels in *Nitrosomonas europaea* and *Methylotetracoccus oryzae*

	Gold particles[a]							
	total	membrane-associated				cytoplasmic	resin	unclear
		total	periplasmic face[b]	cytoplasmic face[b]	unclear[b]			
N. europaea	182 (100)	135 (74)	66 (49)	18 (13)	51 (38)	23 (13)	6 (3)	18 (10)
M. oryzae	174 (100)	106 (61)	75 (71)	8 (8)	23 (22)	28 (16)	3 (2)	37 (21)

[a] Numbers in brackets indicate percentage of total gold particles counted
[b] Numbers in brackets indicate fraction of membrane-associated gold particles (in %)

Table S4. Metagenomic sequencing information for the WWTP sorting experiment. Details on the taxonomic assignment and quality of the retrieved bins, their average coverage, as well as the total number and read percentage aligned to each bin across the different samples are given.

Table S5. *De novo* assembled and binning of the data retrieved from the WWTP sorting experiment. Manually refined bins were matched back to the original bins for naming. Details on the taxonomic assignment of each bin, their average coverage, as well as the total number and read percentage aligned to each bin across the different samples are given.
